# Exploring inclusion complex of an anti-cancer drug (6-MP) with β-cyclodextrin and its binding with CT-DNA for innovative applications in anti-bacterial activity and photostability optimized by computational study†

**DOI:** 10.1039/d2ra05072b

**Published:** 2022-10-28

**Authors:** Modhusudan Mondal, Shatarupa Basak, Salim Ali, Debadrita Roy, Subhadeep Saha, Biswajit Ghosh, Narendra Nath Ghosh, Khusboo Lepcha, Kanak Roy, Mahendra Nath Roy

**Affiliations:** Department of Chemistry, University of North Bengal Darjeeling-734013 India mahendraroy2002@yahoo.co.in vcapdu@gmail.com; Department of Chemistry, Government General Degree College Pedong Kalimpong-734311 India; Department of Chemistry, University of Gour Banga Mokdumpur Malda-732103 India; Department of Microbiology, University of North Bengal Darjeeling-734013 India; Department of Chemistry, Alipurduar University Alipurduar-736121 India; Vice-Chancellor, Alipurduar University Alipurduar-736121 India

## Abstract

The co-evaporation approach was used to examine the host–guest interaction and to explore the cytotoxic and antibacterial properties of an important anti-cancer medication, 6-mercaptopurine monohydrate (6-MP) with β-cyclodextrin (β-CD). The UV-Vis investigation confirmed the inclusion complex's (IC) 1 : 1 stoichiometry and was also utilized to oversee the viability of this inclusion process. FTIR, NMR, and XRD, among other spectrometric techniques, revealed the mechanism of molecular interactions between β-CD and 6-MP which was further hypothesized by DFT to verify tentative outcomes. TGA and DSC studies revealed that 6-MP's thermal stability increased after encapsulation. Because of the protection of drug 6-MP by β-CD, the formed IC was found to have higher photostability. This work also predicts the release behavior of 6-MP in the presence of CT-DNA without any chemical changes. An evaluation of the complex's antibacterial activity *in vitro* revealed that it was more effective than pure 6-MP. The *in vitro* cytotoxic activity against the human kidney cancer cell line (ACHN) was also found to be significant for the IC (IC_50_ = 4.18 μM) compared to that of pure 6-MP (IC_50_ = 5.49 μM). These findings suggest that 6-MP incorporation *via* β-CD may result in 6-MP stability and effective presentation of its solubility, cytotoxic and antibacterial properties.

## Abstract 2

The complexation of an essential anti-cancer drug called 6-Mercaptopurine monohydrate with β-cyclodextrin was investigated for enhancing bioavailability.

## Graphical abstract

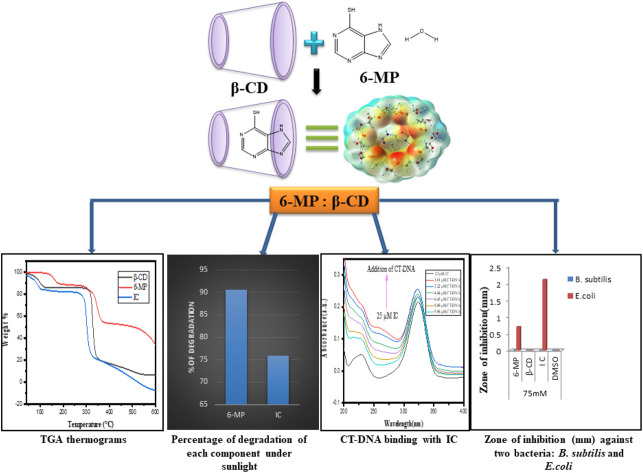

## 1. Introduction

In supramolecular chemistry, cyclodextrins (CDs) are the most intriguing host molecules.^1^ Because of their unique truncated cone structure, they can form stable enclosed complexes (inclusion complexes, IC) with different kinds of guest molecules.^2^ Cyclodextrins have two biphasic layers, one with a hydrophilic peripheral surface and the other with a hydrophobic inner surface. The creation of this sort of stable host–guest encapsulated complex is due to hydrophobic contact between the cavity of the cyclodextrin and the hydrophobic district of the guest molecule. Through various noncovalent interactions (van der Waals force, dipolar interaction, electrostatic and hydrogen bonding), the inner area permits the incorporation of hydrophobic surfaces of different guests or segment(s) of any guest molecules into the cavity of an appropriate and stable geometrical sized CDs.^3,4^ β-Cyclodextrin (β-CD) (Fig. S1†) of 7 glucopyranose units, has been used as the host molecule in this study because of its excellent inclusion efficiency, appropriate cavity dimensions, low cost, and low toxicity.^5,6^ The best way for developing ICs to improve the physicochemical properties of the medicinal molecule is to use cyclodextrin.^7^

6-Mercaptopurine monohydrate (6-MP) (Fig. S1†) is a drug containing adenine analogue that is utilized in the treatment of acute lymphoblastic leukaemia (ALL) in children as well as other kinds of leukaemia.^8^ 6-MP is a potent immunosuppressant and anti-cancer medication that is increasingly used in human and veterinary medicine to treat inflammatory (Crohn's syndrome, ulcerative colitis, rheumatologic illnesses, and so on) disorders.^9^ The United States Food and Drug Administration (FDA) approved this drug just two years afterward the first synthesis. Despite its demonstrated usefulness as an anticancer treatment, 6-MP has certain therapeutic drawbacks. It has low absorption after oral treatment, reaching adequate plasma concentrations after 10–12 hours.^10^ 6-MP has a limited therapeutic index, which means that even the smallest dose increase can produce toxicity. As a result, taking too much 6-MP can induce bone marrow conquest as well as liver issues.^11,12^ As a result, determining the concentration of 6-MP in the human body is critical. 6-MP falls under the biopharmaceuticals classification system (BCS) class II category, which means it has low oral bioavailability (approximately 16%) due to its weak water solubility (0.135 mg mL^−1^),^13^ which limits its biomedical use. Thus, efforts should be undertaken to increase 6-MP's bioavailability and solubility, besides investigating its anticancer effects on specific cancer cell lines, such as lung cancer.

In this article, we have observed the encapsulation of the drug (6-MP) within the nanocavity of β-CD because molecular encapsulation is currently a popular method for increasing the bioavailability of certain medications while maintaining their therapeutic effect. Thus, this technique is capable of improving the pharmacological properties of the encapsulated active substances, such as solubility, chemical stability, and toxicity.^14–16^ As 6-MP is an anticancer drug the anti-cancer activity can also be enhanced and thus it becomes novel treatment techniques.^17,18^ β-CD has been extensively employed as a matrix for medication delivery because it has the potential to modify some undesirable features of incorporated guest molecules. β-CD protects pharmaceuticals from biodegradation; it has high thermal stability, with a breakdown temperature of around 300 °C; it is stable at both basic and neutral pH; and it prevents molecular instability when exposed to oxygen, water, radiation, heat, or internal chemical reactions and also β-CD protects against irritation produced by direct drug entry into the body, as well as incompatibility between pharmaceuticals containing other inactive components.^19,20^ Indeed, the inclusion method has the potential to reduce drug toxicity while increasing drug water solubility. As a result, inclusions can be utilized to deliver medications to the body, transport them to the site of action, and release them consistently throughout time.^21,22^ Thus, controlled release of this drug employing β-CD as the encapsulating agent can reduce the negative effects of 6-MP, and the drug molecule must be able to enter the lipophilic barrier to perform its biological function.^23,24^ β-CD can carry the drug to the physiological site without losing any of its bioactivity^25^ by establishing an inclusion complex and in the domains of applied chemistry and chemical engineering, this assists the realisation of the suggested use of the encapsulated medication.

Furthermore, the antibacterial activity of 6-MP encapsulation was tested against several pathogenic bacteria in order to determine the benefits of 6-MP IC. Lastly, to connect with experimental results, density functional theory calculations are used to evaluate optimal geometries, adsorption energies, non-covalent interaction (NCI), and electrostatic potential energy maps (ESP).

## 2. Experimental section

### 2.1 Materials and methods

TCI, India, provided purists' grades of ≥99% 6-mercaptopurine monohydrate (CAS number 6112-76-1 and molecular weight (MW) 152.18 g mol^−1^), which was utilized without additional purification. A well-known source *i.e.*, Sigma-Aldrich, USA provided β-cyclodextrin of the highest grade ≥0.99% which has the CAS number 7585-39-9 and MW 1134.98 g mol^−1^. The compounds utilized in this study are listed in Table S1;† no additional purification was done.

All of the mother solutions were prepared after verifying the solubility of the 6-MP and β-CD in triply distilled, deionized, and degassed water, and additional strengths of solutions utilized in the experiment were made by mass dilution. On a METTLER TOLEDO AG-285 balance with an ambiguity of ±0.01 mg at 298.15 K, all of the experimental materials were weighed. By adopting adequate precautions, the amount of material lost as a result of evaporation during mixing and working with the solutions was decreased.

A K9 optical TENSIOMETER (made in Krüss GmbH, Hamburg, Germany) was used to detect surface tension with an accuracy of ±0.1 mN m^−1^ using the ring detachment technique by a platinum ring. The temperature of the experimental solutions was kept constant at 298.15 K by passing thermostat water through a double-walled glass jar holding solution.

UV-visible spectra were documented with a wavelength resolution uncertainty of ±2 nm using the instrument JASCO V-530 UV/VIS Spectrophotometer. The measurement temperature was kept persistent through an automatic digital thermostat system.

^1^H NMR spectra in DMSO solvent were acquired at 400 MHz on a Bruker Avance instrument at 298.15 K. Parts per million are used to represent chemical changes.

The KBr disc approach was utilized to record FTIR spectra with a resolution of 0.5 cm^−1^ using a PerkinElmer 10.6.1 FT-IR spectrometer (Shimadzu, Japan). Samples were made as thin KBr discs with a tiny amount of material at room temperature. The scanning range was maintained between 400 and 4000 cm^−1^.

A scanning electron microscope was used to conduct the investigation and collect data on the morphology (JSM-6360). It also looks at the morphological patterns and particle scale of the inclusion complex.

### 2.2 Preparation of inclusion complex

The inclusion complex of 6-MP with β-CD was created by combining 6-MP and β-CD in a 1 : 1 molar ratio using a co-evaporation method.^2,15,23^ Initially, β-CD (102.14 mg, 3.0 mmol) and 6-MP (13.69 mg, 3.0 mmol) were prepared in 30 mL of distilled water separately. The solution of β-CD was then kept at 50 °C for 3 hours with continual stirring upon a magnetic stirrer. Following that, the solution 6-MP solution was judiciously added to the host solution to get our target IC and the reaction was carried out for 24 hours. The aqueous solution used here is 10% ethanol. To attain the pure form, the precipitated product was washed with alcohol and dried in a 100 °C oven. After that, the dry IC is kept in a desiccator at the atmospheric conditions for further analysis.

### 2.3 Encapsulation efficiency calculation

The amount of encapsulated 6-MP found in the solid inclusion complexes was quantified using UV-visible spectrophotometry according to the procedures of Kfoury *et al.*^26,27^ 1 mg of the complex was dissolved in 10 mL of distilled water, sonicated at room temperature for 10 minutes, then centrifuged for 5 minutes at 4000 rpm. The encapsulated 6-MP was discharged into the aqueous solution from the cavity of the β-CD. After that, JASCO V-530 UV/VIS spectrophotometer was used to measure the 6-MP concentration at 324 nm. The calculation of the EE percent was done using the following formula:

1 EE% = {6-MP_exp_ (mg)/6-MP_*t*_ (mg)} × 100

where 6-MP_*t*_ denotes the amount of theoretical 6-MP weighed for the formation of the solid complex, and 6-MP_exp_ denotes the quantity of experimental 6-MP in the lyophilized complex, as measured spectrophotometrically.

### 2.4 Aqueous solubility measurement

According to the references,^28,29^ the solubility of 6-MP-β-CD in water was determined at a temperature of 25 °C. To measure the aqueous solubility of the IC, we have prepared seven set of test solutions having different concentrations. After that, the UV-Vis spectrum of each solution was taken using JASCO V-530 Spectrophotometer. Then a plot of absorbance against concentrations of IC provides a straight line as shown in Fig. 10. Finally using the formula of Lambert-Beer law—*A* = *εcl*, where *A* is the absorbances of the solutions, *ε* denotes the absorption co-efficient, *c* defines the concentration and *l* is the path length. The absorption coefficient was evaluated as 0.8236 L g^−1^ cm^−1^ and the solubility of the inclusion complex was also determined accordingly.

### 2.5 Antimicrobial activity study

The antimicrobial activity of 6-MP was tested in relation to the bacteria *Bacillus subtilis* and *Escherichia coli* by well diffusion assay.^15,30^ Briefly, the bacterial cultures were grown overnight in Mueller Hinton broth and uniformly spread on the surface of Mueller Hinton Agar plates using sterile cotton swabs. Wells of diameter 8 mm were made using sterile cork borers and 6-MP and β-CD were used singly and in combination (in the form of inclusion Complex IC) at concentrations of 75 mM. The plates were incubated overnight at 37 °C and observed for the formation of inhibition zones. The “diameter of the inhibition” for positive results was estimated by subtracting the diameter of the well (8 mm) from the diameter of the zone of inhibition.^15^ The diameter of inhibition when no zone was observed was kept at zero. In this study, Ampicillin as positive control and sterile distilled water as negative control in both *B. subtilis* and *E. coli* have been taken and provided in ESI file (Fig. S2†).

### 2.6 MTT assay

A 96-well microtiter plate was used to grow ACHN (Human Malignant Kidney Cell Line), which was obtained from NCCS in Pune, India. The cell line was grown at a density of 4 × 10^3^ cells per well in 100 μL of DMEM (Dulbecco's modified eagle medium) Ham F-12 growth medium at 37 °C in the presence of 5% CO_2_.^15^ After a 24-hours incubation period, different doses of the drugs 6-MP and its IC (50 μM to 500 μM) were added in triplicate to each well. The microtiter plate was incubated once more under identical experimental circumstances. The treated plate was taken out of the incubator after the culture media had been discarded, and 10 μL (5 mg mL^−1^) of MTT powder diluted in 1× PBS was applied to each well. The plate was then kept in the same condition for three hours. Finally, 50 μL of isopropanol, a formazan solubilizer, was added to each well containing MTT solution and stirred for about 5 minutes. The absorbance at 620 nm was then measured using an ELISA reader. The mean optical density of untreated cells (*X*) and the mean optical density of treated cells (*Y*) at various drug dosages were used to calculate the fraction of cell toxicity ((*X* − *Y*)/*X* × 100).^15,23^

### 2.7 Computational details

All the simulations in the present work have been done employing density functional theory (DFT) using the Gaussian 16 program.^31^ Ground state geometry optimizations of the 6-MP, β-CD and 6-MP-β-CD inclusion complex were carried out at the M06-2X/6-31+G(d) level of theory.^15,23^ Previous studies report that compared to other hybrid functionals, the meta-generalized gradient approximation M06-2X functional provides reliable and precisely non-covalently bonded interaction energies (hydrogen bonding, π–π stacking) in non-covalently bonded systems.^32^ Eventually during optimization solvent effects (water) were incorporated by applying the polarizable continuum model (PCM)^33^ using the integral equation formalism variant. To check whether the optimized geometry resides to the minima on the potential energy surfaces, vibration frequency analysis (no imaginary frequency) was done at the same level of theory.^34,35^ To analyse weak interactions like H-bonding, van der Waals interactions, steric interactions non-covalent interaction (NCI) index plots of the reduced density gradient (RDG *or s*) *vs.* molecular density *ρ* were plotted using the Multiwfn 3.7 suite^36^ with the ground state geometries. Charge densities and molecular electrostatic potential (MESP) maps were plotted at the same level of theory to visualize the extent of existing charge-transfer interactions in the 6-MP-β-CD inclusion complex in aqueous medium. Finally, utilizing the following formula, adsorption energies or binding energies (Δ*E*_ads_) of the complex systems were calculated:

2 Δ*E*_ads_ = *E*_6-MP-β-CD_ − *E*_6-MP_ − *E*_β-CD_

where *E*_6-MP-β-CD_, *E*_6-MP_, and *E*_β-CD_ are the total energy of the geometry optimized complexes, free 6-MP and the β-CD molecules, respectively.

## 3. Result and discussion

### 3.1 Job plot confirms the stoichiometric ratio

**Fig. 1:**
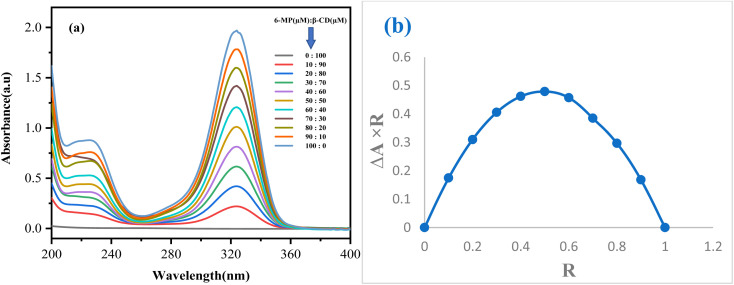
Generation of Job plot (a) spectra and (b) the obtained Job plot.

The stoichiometry of the IC generated can be determined using Job's approach. UV-visible spectroscopy is used to create job plots using the continuous variation method.^15,37,38^ A series of solutions for the drug 6-MP and both β-CD were created for this purpose, with the mole fraction of the drug molecule varying (Fig. 1a). The difference in the absorbance of the drug without and with the presence of host β-CD (Δ*A*) × *R* was plotted against *R* to get these plots, where, Δ*R* = [6-MP]/([6-MP] + [β-CD]). At 298.15 K, absorbance values for each of the solutions were recorded at their respective peaks (Table S2†). The stoichiometry of the IC was evaluated using *R* values at maximum deviation, *i.e.*, the ratio of host to guest at *R* = 0.5 for a 1 : 1 complex, *R* = 0.33 for a 1 : 2 complex, and *R* = 0.66 for a 2 : 1 complex.^23,39^ The maxima for each plot have been discovered to be at *R* = 0.5, confirming the 1 : 1 stoichiometry between 6-MP and both β-CD to form IC (Fig. 1b).

### 3.2 Surface tension investigation validates the inclusion with stoichiometric ratio

**Fig. 2:**
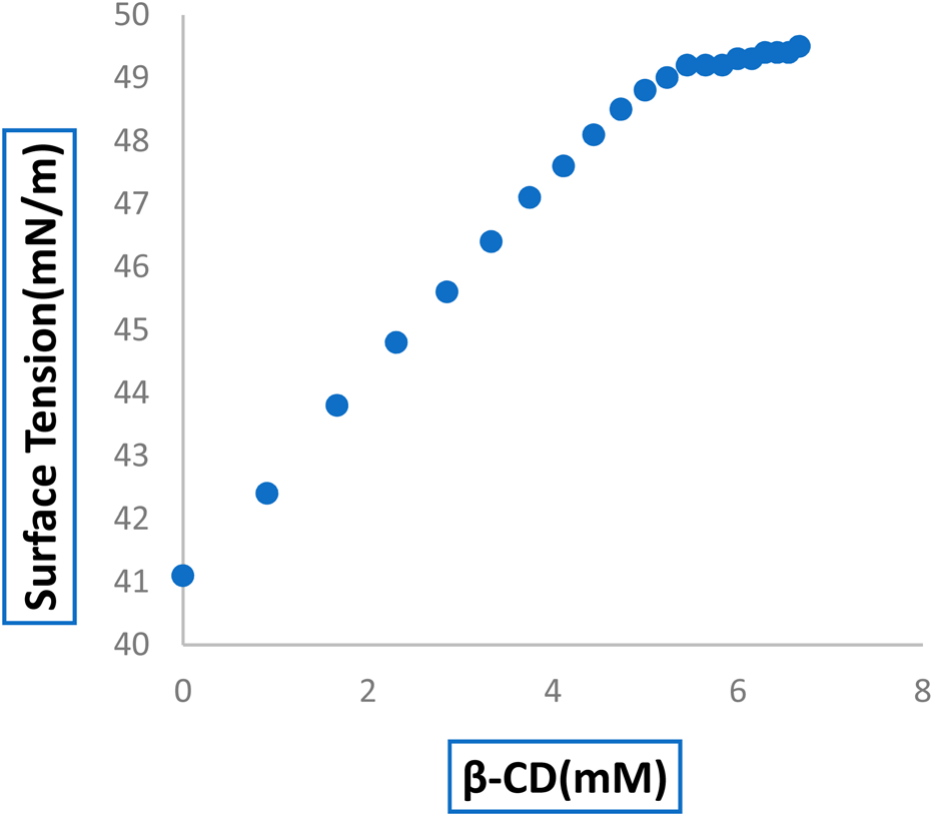
Variations in the surface tension of the 6-MP with increasing concentration of (a) β-CD.

Surface tension (*γ*) analysis can be used to determine the development of the host–guest IC and its stoichiometry.^23,40^ Because of the limited water solubility of 6-MP, 6-MP and β-CD solutions were made using 3 : 4 (v/v) AcN : H_2_O as the solvent in this investigation. The surface tension of the 6-MP solution was found to be lower than that of pure 3 : 4 (v/v) AcN : H_2_O, indicating that 6-MP is surface active, which could be due to the presence of a hydrophobic indole moiety.^41,42^ When dissolved in a significant range of concentrations, β-CD with hydrophilic rims and hydrophobic external surfaces do not show any change in the surface tension of the ratio of 3 : 4 (v/v) AcN : H_2_O.^43,44^ In our investigation, we discovered that the surface tension of the 6-MP solution increased as the concentration of both β-CD molecules increased, implying that surface-active 6-MP molecules have been incorporated into the vital hydrophobic hollow of β-CD molecules to produce IC in the solution phase (Table S3†).^41^ A break point appeared in the figure, indicating the formation of IC as well as its 1 : 1 stoichiometry (Fig. 2). Table 1 shows the concentration of 6-MP and β-CD at the breaking point, along with the associated value. The breakpoint originated at a concentration where the molar concentration ratio of the studied host β-CD to the guest 6-MP molecule is almost 1 : 1.^45^

**Table 1:** The surface tension (*γ*) values at the break point and the corresponding β-CD and 6-MP concentrations

Host molecules	Conc. of 6-MP (mM)	Conc. of β-CD (mM)	Surface tension (*γ*) mN m^−1^
β-CD	5.06	4.94	48.89

### 3.3 Association constant and Gibbs energy parameter

**Fig. 3:**
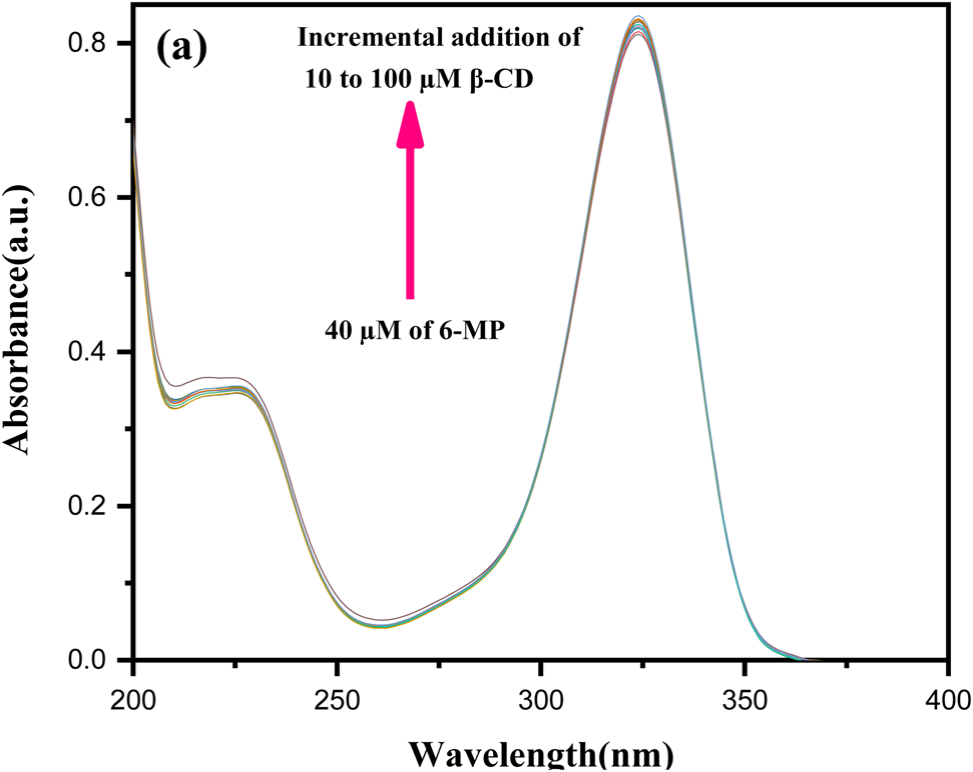
Absorption spectra of 6-MP (40 μM) in various concentrations of aqueous (a) β-CD in μM.

Using UV-Vis spectroscopy,^15,23^ the estimated formation constant (*K*_a_) of the IC in the solution state was used to investigate the non-covalent binding capability of the 6-MP molecule within the cavity of β-CD, as well as the binding strength of the corresponding IC.^46^ The molar extinction coefficient (*ε*) of 6-MP's chromophore was shifted as it transitioned from a polar aqueous medium to an apolar cavity of CDs *via* non-covalent interactions to generate host–guest IC as the solvent polarity changed. The change in absorbance (Δ*A*) of 6-MP at *λ*_max_ = 324 nm was measured (Table S4†) by gradually raising the concentrations of β-CD by adjusting the temperature at 298.15 K for the estimation of *K*_a_ (Fig. 3).^47–49^ The Benesi–Hildebrand method was used to generate the double reciprocal plot for 1 : 1 6-MP-β-CD (host–guest) complexation, and the related eqn (3) is as follows:3
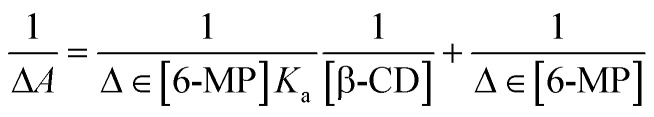
where Δ*A* is the difference in 6-MP absorbance at maximum wavelength and Δ*ε* is the difference in 6-MP molar extinction co-efficient from polar to the apolar environment. The plot is a straight line and the *K*_a_ for the IC is calculated from the intercept to the slope of the double reciprocal plot's straight line (Fig. S3†). The association constant value determines the guest's capacity to bond to the host. The higher the value of the association constant, the greater the stability of the IC. The IC formed with β-CD was found to be more stable as 6-MP strongly binds with the proper binding site of the hollow cavity of the host molecule.

The change in free energy (Δ*G*) was calculated once more using the well-known stability constant-free energy relationship (eqn (4)).

4 Δ*G* = −*RT* ln *K*_a_

where Δ*G* denotes the change in free energy, *K*_a_ is the association constant, *R* denotes the universal gas constant, and *T* denotes the temperature presented in Kelvin (K). The change in Gibbs free energy for the (6-MP-β-CD) system was exposed to be negative, implying that the formation of the complex (IC) *via* interactions is possible and thermodynamically plausible^15,50,51^ (Table 2). Accordingly, the free energy value, Δ*G*, directing spontaneity in the formation of IC as well.^15^

**Table 2:** Values of association constant (*K*_a_) and free energy change (Δ*G*^0^) of the (6-MP-β-CD) system

Complex systems	*T* a/*K*	*K*_a_/M^−1^	Δ*G*^0^/kJ mol^−1^
(6-MP-β-CD)	298.15	7.98 × 10^3^	−22.26

a Standard uncertainty in temperature is: (*T*) = ±0.01 K.

### 3.4 Encapsulation efficiency (EE%) determination

The amount of drug trapped in the cavity was determined by measuring the encapsulation efficiency using a carrier solvent *i.e.*, the β-CD inclusion abilities toward 6-MP were assessed spectrophotometrically, as indicated in Table 3. The IC that was produced utilising β-CD and 6-MP at a 1 : 1 molar ratio had the EE = 87.1 ± 2.4%. The binding capacity may be increased by 6-MP inserting more deeply into β-CD. These findings may be explained by the fact that CDs have a propensity to aggregate. The larger EE in complexes may be attributed to the 6-MP being adequately trapped inside the interior cavity of the β-CD and noncovalent interactions such as dipole–dipole, van der Waals, and hydrophobic contacts between them. This is the first time that the encapsulation of 6-MP into β-CD has been documented, to our knowledge.

**Table 3:** Encapsulation efficiency (EE%) β-CD inclusion ability toward 6-MP

Inclusion complex	Encapsulation efficiency (EE%)
β-CD/6-MP (1 : 1)	87.1 ± 2.4

### 3.5 ^1^H NMR spectra investigation

**Fig. 4:**
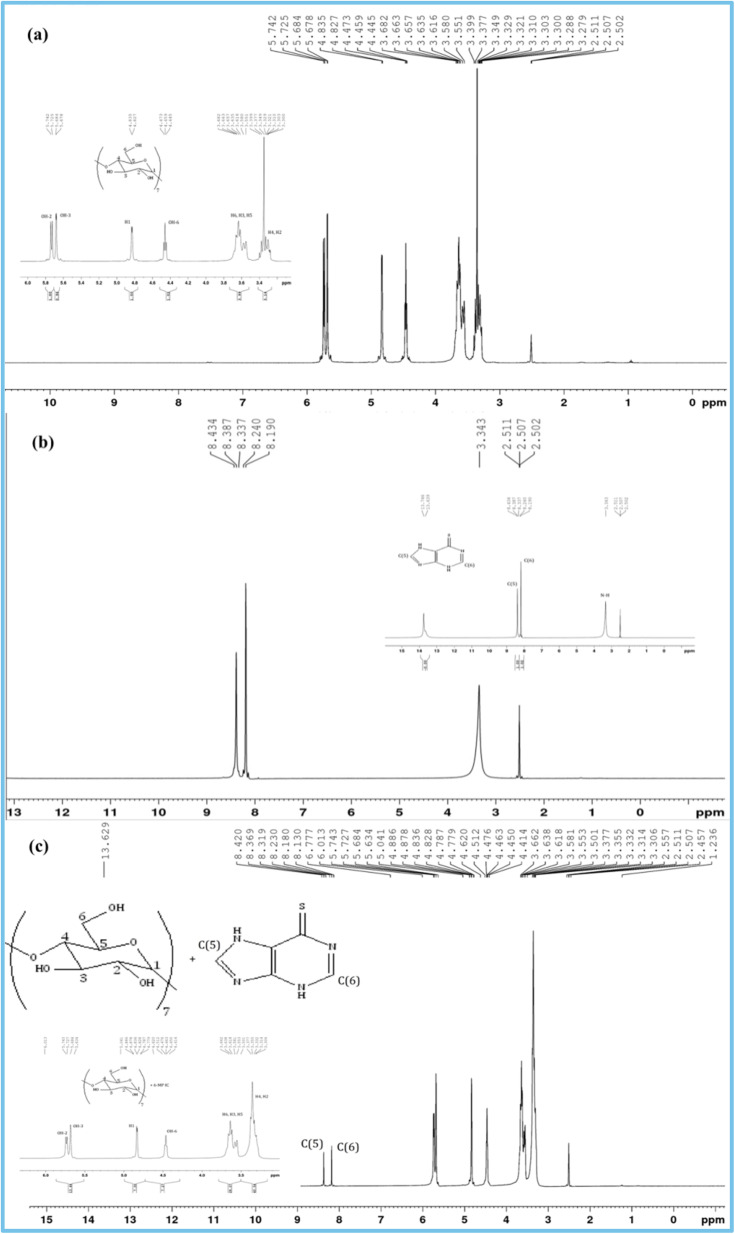
^1^H NMR spectra of (a) β-CD, (b) 6-MP, and (c) IC.

^1^H NMR research confirms the formation of IC and its stoichiometry.^15^ The shift of signals of the protons of 6-MP and β-CD molecules occurs when the drug molecule is enclosed inside the hydrophobic cavity of the studied host β-CD^52,53^ (Fig. 4). The interaction with the β-CD protons after encapsulation causes the aromatic part of the 6-MP molecule to produce diamagnetic shielding of protons present in it. In Fig. S1,† the position of several protons in β-CD molecules is depicted. H3 and H5 protons are found near the larger and finer rims of the CD cone, respectively.^54–57^Tables 4 and 5 list the chemical shift (*δ*) values for the drugs 6-MP, β-CD, and their IC. Table 4 of NMR spectra clearly shows the upfield shift of H3 and H5 protons of β-CD, demonstrating the formation of IC with the 6-MP molecule and indicating that the drug molecule enters the hydrophobic cavity from the wider rim. As shown in Table 4, it is also well established that the chemical shift values of H1, H2, H4 and H6 protons of β-CD with or without 6-MP are almost same because they are located on the external surface.^58^ The aromatic protons of 6-MP show significant downfield shifts, confirming the encapsulation of the 6-MP molecule inside the cavity of the β-CD rings.^59,60^ These findings demonstrate the formation of the IC.

**Table 4:** Chemical shift Data (in ppm) of protons of β-CD in Free State and during IC formation with 6-MP

Protons of β-CD	*δ* (free) (ppm)	*δ* (complex) (ppm)	Δ*δ* (difference) (ppm)
H3	3.663	3.618	0.045
H5	3.565	3.527	0.038
H1	4.831	4.832	0.001
H2	3.303	3.314	0.011
H4	3.399	3.377	0.022
H6	3.682	3.662	0.020
OH-2	5.733	5.735	0.002
OH-3	5.681	5.684	0.003
OH-6	4.459	4.463	0.004

**Table 5:** Chemical shift data (in ppm) of protons of 6-MP in free state and during IC formation with β-CD

Protons of 6-MP	*δ* (free) (ppm)	*δ* (complex) (ppm)	Δ*δ* (difference) (ppm)
C–H (6 membered ring)	8.190–8.240	8.130–8.230	0.035
C–H (5 membered ring)	8.337–8.434	8.319–8.420	0.016

### 3.6 FTIR spectra study

**Fig. 5:**
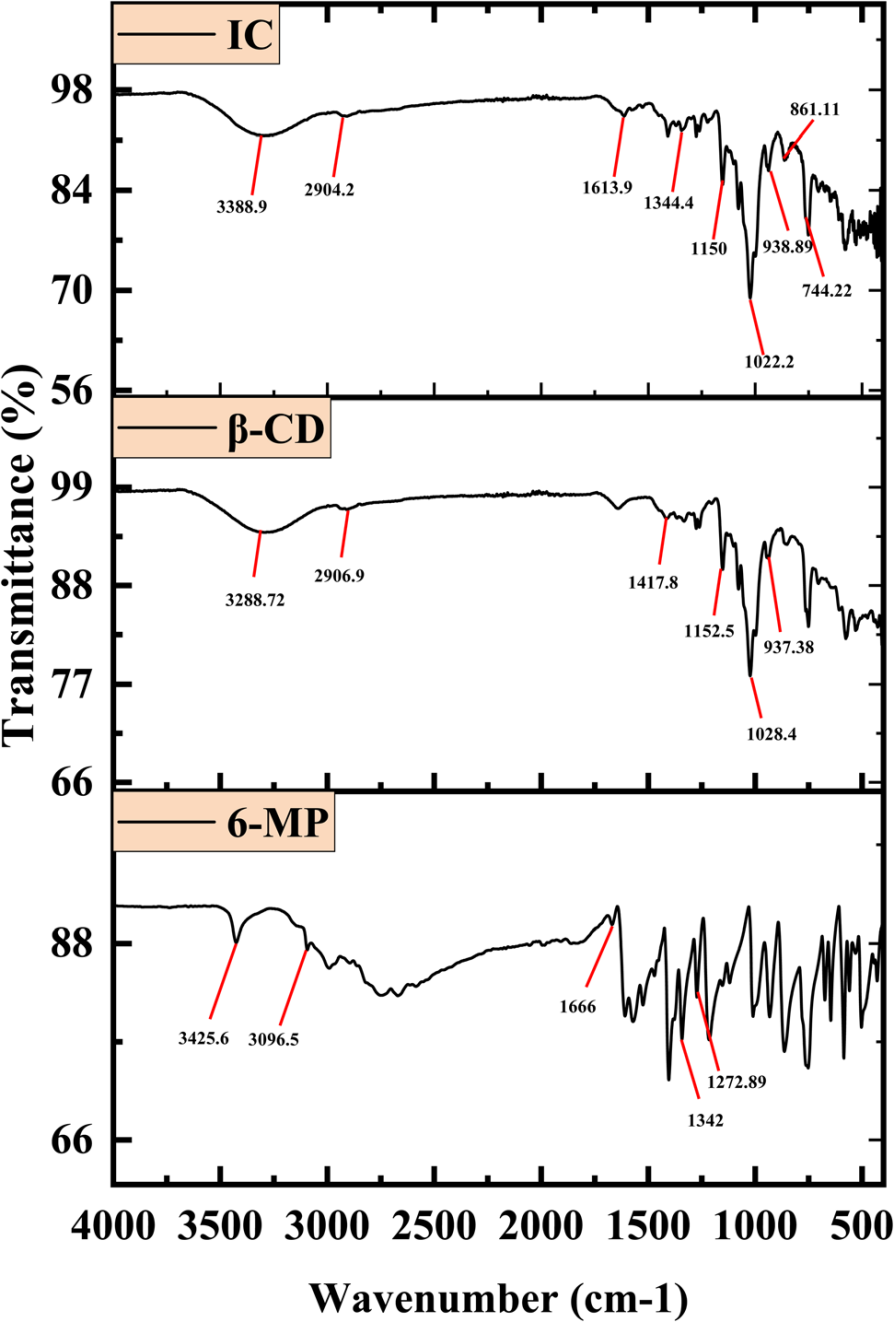
FTIR spectra of 6-MP, β-CD, and 6-MP-β-CD IC.

The interaction between the 6-MP and β-CD in the IC can be determined *via* FT-IR spectrum analysis.^23,61,62^ Excluding covalent interactions, the hydrophobic contacts, van der Waals interactions, and hydrogen bonding are responsible for the drug 6-MP and the β-CD forming IC in the solid state. The resulting absorption bands from the enclosed region of the guest have been altered in their position after the drug molecule was encapsulated by the host β-CDs.^63,64^Fig. 5 shows the spectra, and Table S5† lists the characteristic signals, along with the chemical bonds responsible for the associated stretching and bending frequencies. The shifting of IR signals may be used to re-establish the encapsulation modes of 6-MP into β-CD, as shown by NMR investigations.^65,66^ The formation of IC makes many changes in spectra than those of 6-MP and β-CD. The signal of stretching of –C–H from –CH_2_ of β-CD was at 2906.90 cm^−1^, whereas the IC showed a signal at 2904.20 cm^−1^ may be due to the close proximity of –C–H of β-CD with 6-MP. Stretching of C=C showed signal at a 1666.00 cm^−1^ for pure 6-MP, which changed at 1613.90 cm^−1^ for the IC possibly owing to interaction with β-CD cavity. Stretching of C–N of pure 6-MP was at 1342.00 cm^−1^, which shifted for the IC at 1344.40 cm^−1^ possibly because of the formation of IC between β-CD and 6-MP. Stretching of –C=S of free 6-MP was at 1272.89 cm^−1^, which shifted for the IC at 1150.00 cm^−1^ due to encapsulation into the cavity of CD. Stretching of –C–C–O of β-CD was at 1028.40 cm^−1^, which shifted for the IC at 1022.20 cm^−1^ due to close proximity of β-CD with 6-MP.

The spectra of the ICs show no extra peaks. This fact confirms that the host and guest have only non-covalent interactions and that only van der Waals interactions exist.

### 3.7 Structural influences of oligosaccharide

The structural combination of both molecules is important to the creation of host–guest IC.^23^ Only when the guest and host molecules have a strong interaction than other forces do ICs form. The strength of the encapsulation is determined by parameters such as the guest molecule's size, van der Waals interactions, charge transfer interaction, water molecule release, hydrophobic interaction, hydrogen bonding, and the energy release due to conformational strain. During the formation of IC, the hydrophobic component of the drug (6-MP) molecule enters the hydrophobic hollow of the β-CD molecule, and no covalent link is formed or broken in the IC system.^67,68^

**Fig. 6:**
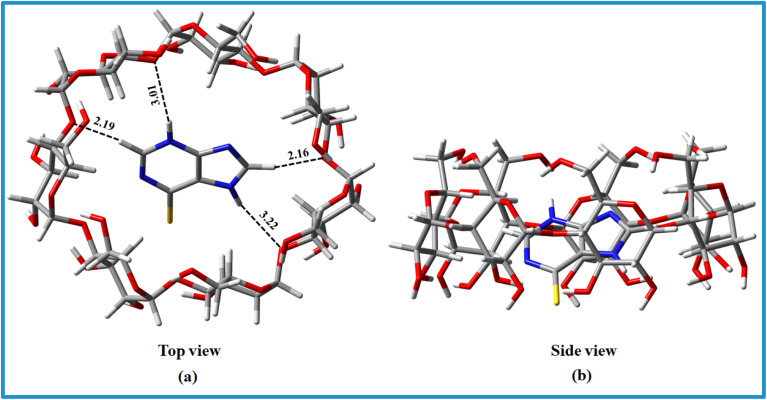
Top and side views of the optimized geometries for the 6-MP-β-CD complex at M06-2X/6-31+G(d) level of theory. Red, gray, white, blue and yellow color represents oxygen, carbon, hydrogen, nitrogen and sulfur atoms respectively.

Water molecules block the cavity of the β-CD molecule, which is an unfavourable situation. The water molecules are easily replenished when the hydrophobic region of the guest molecule enters the hollow chamber of the β-CD. The hydrophobic interaction is dominant in this aspect. However, when the H_2_O molecules are eliminated to bulk, the system's entropy increases, which contributes to the spontaneous development of ICs. Another important factor for the formation of the IC is the size of the 6-MP as a guest molecule, or more precisely the hydrophobic province of the guest molecule that comes in the β-CD cavity (Fig. 6). The hydrophobic component of 6-MP fits better within β-CD by dropping the β-CD's ring strain and lowering the system's energy. The UV-Vis study of the Job plot reveals that encapsulating a single 6-MP molecule sterically blocks the side of the larger rim, preventing further molecules from entering the cavity.

### 3.8 SEM images and surface morphology

**Fig. 7:**
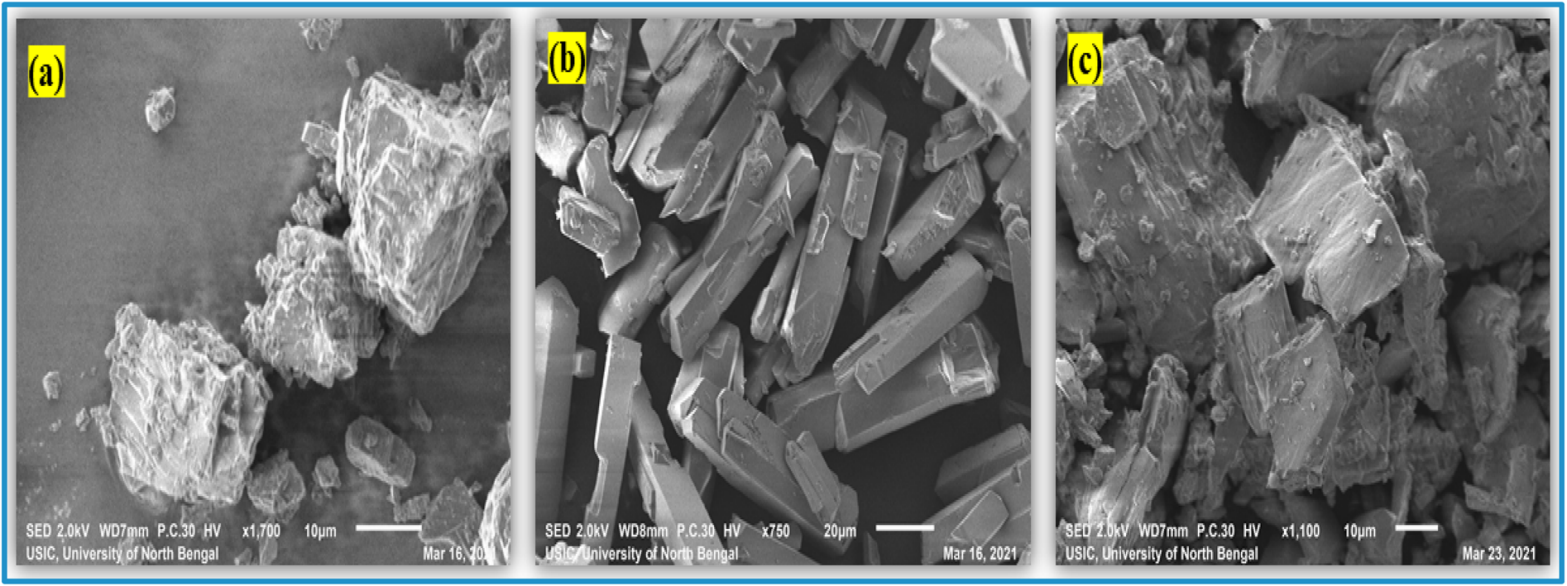
SEM images of (a) β-CD, (b) 6-MP, and their (c) IC.

The images, to be precise, the surface character, morphology, and particle size of solid samples can all be investigated using scanning electron microscopy.^69,70^ This also contributes to our knowledge of how ICs occur between the 6-MP and β-CD molecules.^23^ The surface morphology of pure 6-MP, β-CD, and the resulting ICs are shown in Fig. 7. On SEM pictures, the individual pure β-CDs are visible with their cubic type size, whereas the 6-MP is comparably quite long rectangular flakes type crystal in structure and visible clearly with their unique shape. On the other hand, the (6-MP-β-CD) inclusion complexes formed are relatively crystals in nature with morphological surfaces that are completely different from each 6-MP and β-CD. Fig. 7 shows that the surface roughness and size of the individual host and guest molecules, as well as the ICs generated, are completely different. Furthermore, the presence of β-CD complexation confirms the development of 6-MP inclusion complexes with β-CD, which could complement the findings of ^1^H NMR research.

### 3.9 Powder XRD analysis

**Fig. 8:**
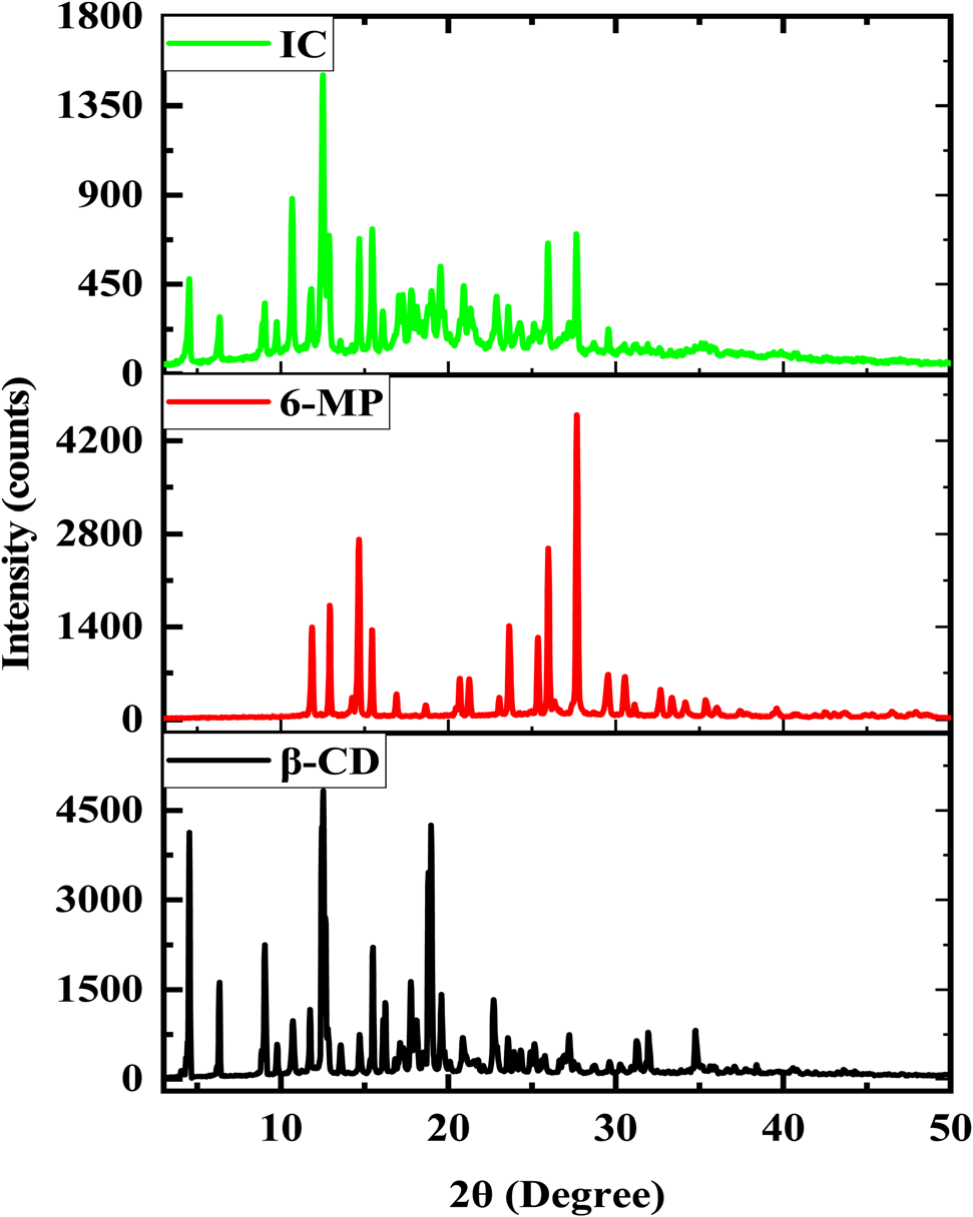
PXRD spectra of β-CD, 6-MP and its IC.

Powder XRD diffraction spectrum analysis may offer direct evidence for the detection of β-CD complexation with 6-MP in powder states because the crystalline form of guest molecules has been altered in the solid phase.^71,72^ By comparing the diffraction outlines of the pure sample and the generated complex sample, the formation of the target solid IC may be proven. The diffraction forms of β-CD, 6-MP, and their IC are shown in Fig. 8. The distinctive peaks for β-CD are at 4.53, 6.31, 9.03, 12.44, 12.53, 12.65, 15.49, 17.76, 18.81, 18.97, and for 6-MP at 11.85, 12.91, 14.65, 15.45, 23.63, 25.35, 25.96, 27.66 respectively.^73^ Due to the encapsulation of 6-MP, the sharp and powerful peaks of β-CD and 6-MP that reveal their crystalline property are moved, erased, or become less intense in complex forms. The IC showed the different peaks at 4.53, 6.35, 9.03, 10.67, 11.81, 12.51, 12.87, 14.67, 15.47, 16.11, 17.07, 17.27, 17.82, 19.01, 19.54, 20.93, 22.90, 23.59, 25.94, 27.66. We also observed that the solid IC produced by pure guest 6-MP and pure host β-CD did not superimpose with the above peaks. The contrasts between these patterns indicate the emergence of a new solid phase between 6-MP and β-CD evidenced the formation of IC as well.^74,75^

### 3.10 DSC study

**Fig. 9:**
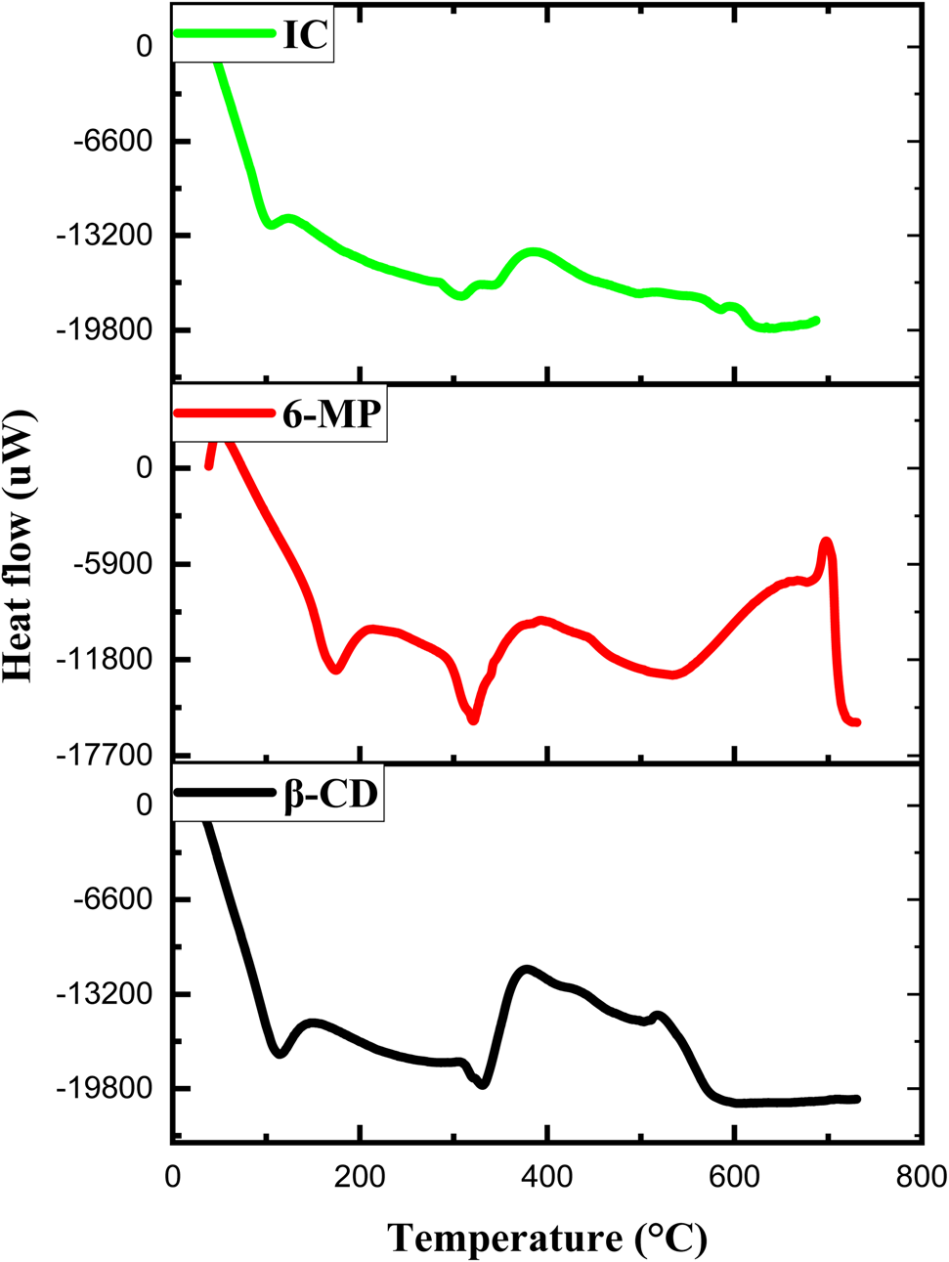
DSC thermograms of β-CD, 6-MP and its IC.

The IC was characterized to find out the thermal properties using the DSC technique, and the data was then used to provide additional evidence for the production of the IC.^76^ Due to the development of IC, the properties of the pure compound, like its unique boiling, melting, or sublimation point frequently vanish or move to different temperatures.^77,78^Fig. 9 depicts the DSC thermograms of β-CD, 6-MP and its IC. There was wider endothermal peak associated with loss of water for β-CD at about 110 °C and 326 °C, whereas 6-MP showed the endothermic peaks at 170 °C and 319 °C. However, in the DSC thermogram of IC, endothermic peaks at around 100 °C and 300 °C with a lower strength were observed. The peak at 300 °C for IC suggests that the 6-MP molecule has lost its crystalline form, as well as indicating that the 6-MP molecule has significant interaction with β-CD. From a thermal standpoint, these verdicts support the formation of IC between 6-MP and β-CD.

### 3.11 TGA analysis

**Fig. 10:**
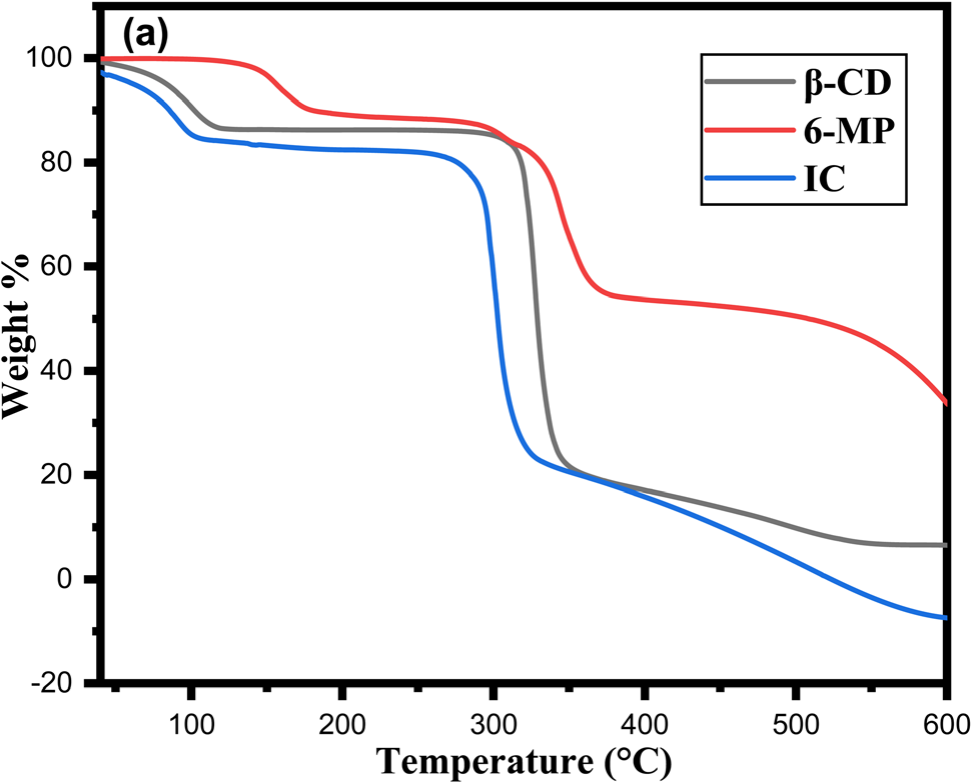
(a) TGA of β-CD, 6-MP and its IC.

Thermal analysis is a key tool for determining the decomposition nature of the inclusion complex.^79^Fig. 10a shows the thermos analytical curves of TGA for 6-MP and IC. According to prior research, β-CD has two phases of mass loss in TGA; the first occurred between 34 °C and 107 °C, indicating the loss of water molecules.^80^ The second stage of mass loss, which was more intense, occurred between 297 °C and 352 °C and was caused by the disintegration of the β-CD structure as a result of the transition from solid to liquid phase.^81^ However, 6-MP revealed three-step weight loss from the TG curve at 118–165 °C, 290–370 °C and 515–700 °C which were related to the disintegration of 6-MP. The weight loss of 6-MP in the IC occurred in three-phase fashion. First, a small step from 34–96 °C, then sharp between 271-324 °C and lastly continuous up to 590 °C, corresponds to the weight loss of 6-MP during heating, indicating the formation of the 6-MP-β-CD complex. A high peak at 327 °C on the DTG curve of β-CD alone could be the consequence of β-CD deterioration. The endothermic peak of pure 6-MP, which formed at 343 °C, completely replaced with the new endothermic peak in IC at 301 °C in the DTG curve (Fig. S4†). When compared to pure 6-MP, the peak that appeared in the IC was different, proving that 6-MP had been entirely encapsulated in the nanocavity of β-CD.

### 3.12 Solubility study

**Fig. 11:**
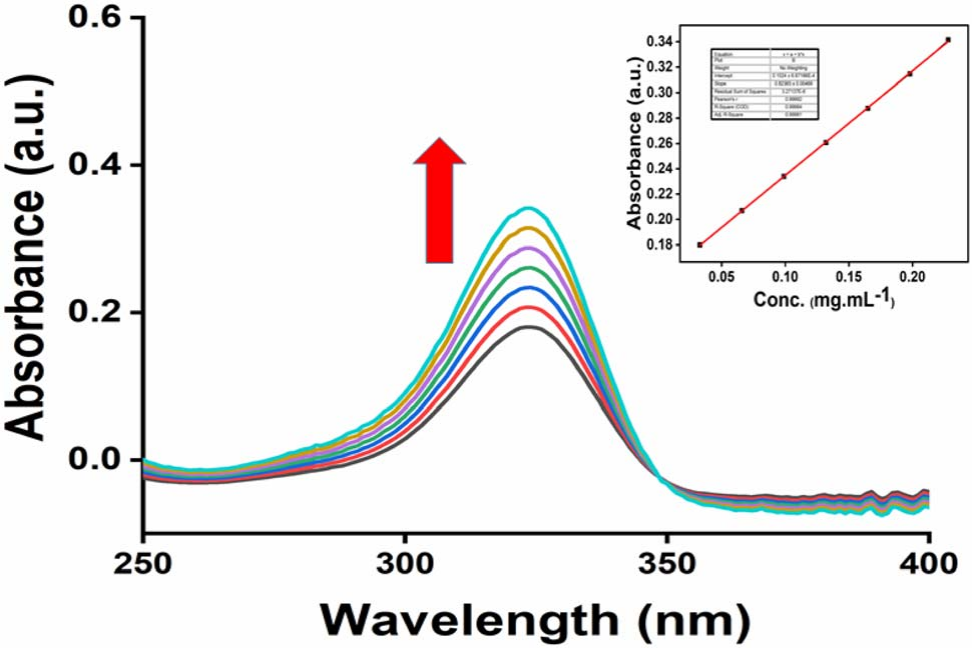
UV-spectra of 6-MP+β-CD of different concentrations (mg mL^−1^) in aqueous medium (a) 0.033, (b) 0.066, (c) 0.099, (d) 0.132, (e) 0.165, (f) 0.198, and (g) 0.228.

**Fig. 12:**
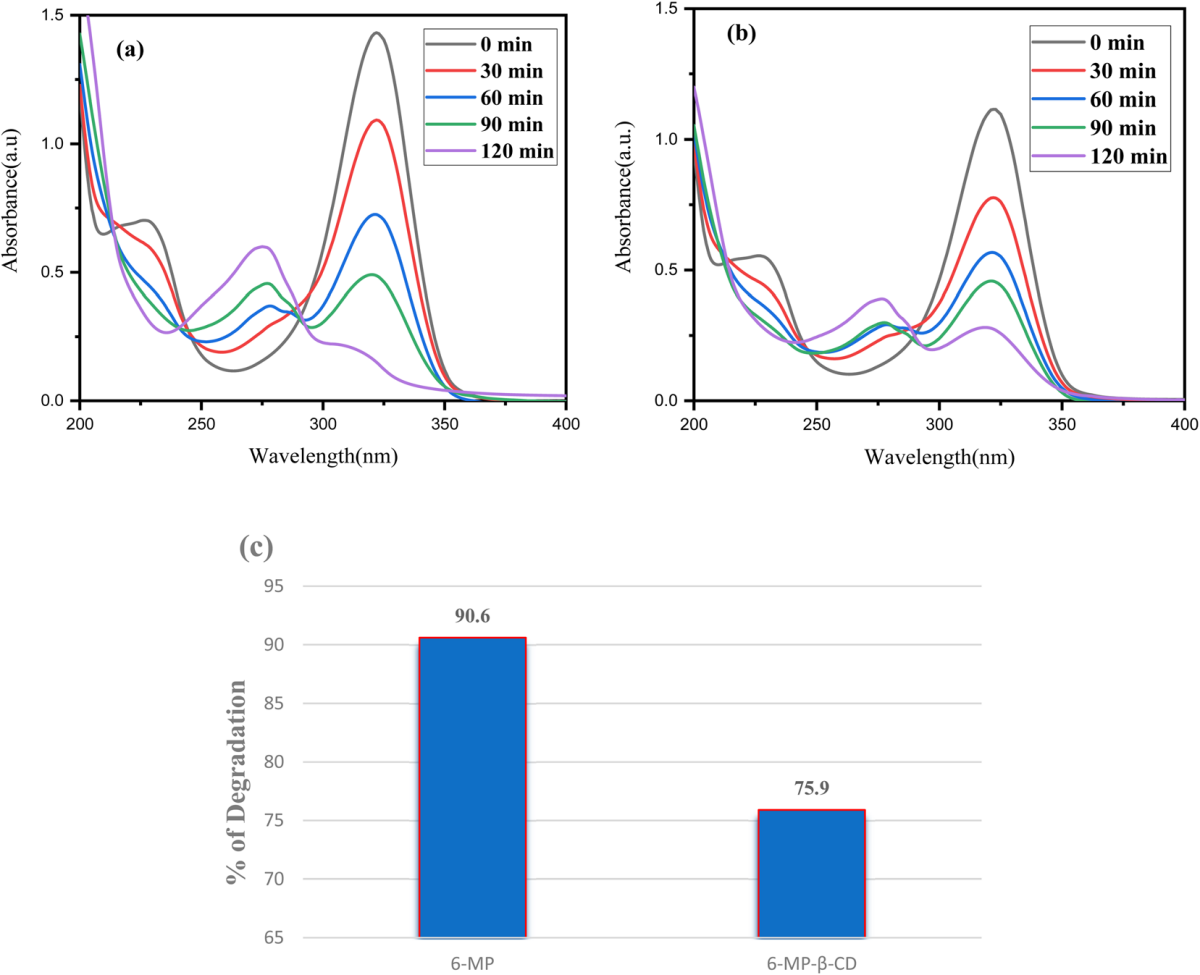
Time-dependent UV-Vis spectra of (a) 6-MP, (b) IC and (c) percentage of degradation of each component under sunlight.

UV-Vis spectroscopy can be used to compare the water solubilities of pure 6-MP and IC.^82,83^Fig. 12 depicts the UV-Vis spectrum of the IC in aqueous solution (pH = 7.0, 25 °C). 6-MP was very weakly soluble in water; however, the formation of the IC substantially increased the solubility of 6-MP in water. Only the peaks of 6-MP could be detected in the inclusion complex since β-CD did not absorb in the UV-Vis spectrum. According to Fig. 11, the IC showed an absorption peak at a wavelength of about 324 nm. The peak positions did not depend on the 6-MP+β-CD concentrations. However, it was noted that the peaks intensity increased with increasing the concentrations of 6-MP+β-CD. The graph of IC absorbance at 324 nm *vs.* concentration yields a straight line, as shown in Fig. 11 (inside). The absorption coefficient of IC in aqueous solution (pH = 7.0, 25 °C) was calculated as 0.8236 L g^−1^ cm^−1^, in accordance with the Lambert–Beer law. Fig. S5† displays the UV spectra of the IC at saturation concentrations in aqueous solution (pH = 7.0, at 25 °C). The saturated solution of the IC's absorbance value was determined to be 1.3687 (Fig. S5†). Table 1 shows the solubilities of pure 6-MP and IC in water at 25 °C. It is evident from Table 1 that IC is more soluble in water than pure 6-MP. These findings demonstrated that the development of the IC significantly increased the solubility of 6-MP in aqueous solution (Table 6).

**Table 6:** Aqueous solubility of 6-MP and 6-MP+β-CD at 25 °C

Sample	Solubility	Ref.
6-MP	0.135 mg mL^−1^	13
IC	1.661 mg mL^−1^	In this study

### 3.13 Photostability study

When exposed to sunshine, the photodecomposition of both the free medication 6-MP and the 6-MP-β-CD IC was investigated.^15^ To compare the photostability of IC to that of pure 6-MP, we conducted photodegradation experiments by comparing the UV spectra of both the guest and IC complex before and after exposure to sunshine, and we found a shift in spectral intensity.^84^ We prepared a 100 μM 50 mL solution of both the 6-MP and the IC and took in two individual 100 mL beakers, respectively. The adsorption–desorption equilibrium was then achieved by magnetically stirring these two reaction mixtures for 30 minutes. The photodecomposition performance of the reaction mixtures was next tested by exposing them to visible sunlight (Fig. 12). Following the photo decomposition experiment, 3.5 mL aliquots of each solution were extracted at a predetermined time interval to determine their concentration in terms of absorbance change at 324 nm in a UV-Vis spectrophotometer. The proportion of degradation (percentage) was calculated using the following equation:

5 % of degradation= (*A*_0_ − *A*_*t*_) × 100/*A*_0_

For each process, *A*_0_ is the drug solution's initial absorbance, and *A*_*t*_ is the drug solution's ultimate absorbance after time *t* (in minutes). When these two values are compared, it can be shown that pure 6-MP degrades more than 6-MP-CD IC when exposed to sunshine. The degradation percentage for pure 6-MP was determined to be 90.60%, while the degradation percentage for IC was only 75.94% in 120 minutes (Fig. 12c). This result suggests that the photostability of 6-MP-CD IC is higher than that of pure 6-MP, demonstrating the usefulness of CD complexation in increasing the photostability of 6-MP. The improved photostability of IC can be related to the host–guest interaction, where β-CD protects 6-MP and lowers photodegradation effects when compared to the free drug.^15,85^ Thus, this study supported the stability of the β-CD-6-MP molecular assembly.

### 3.14 CT-DNA binding study: the procedure for discharging a guest from the cavity of β-CD

**Fig. 13:**
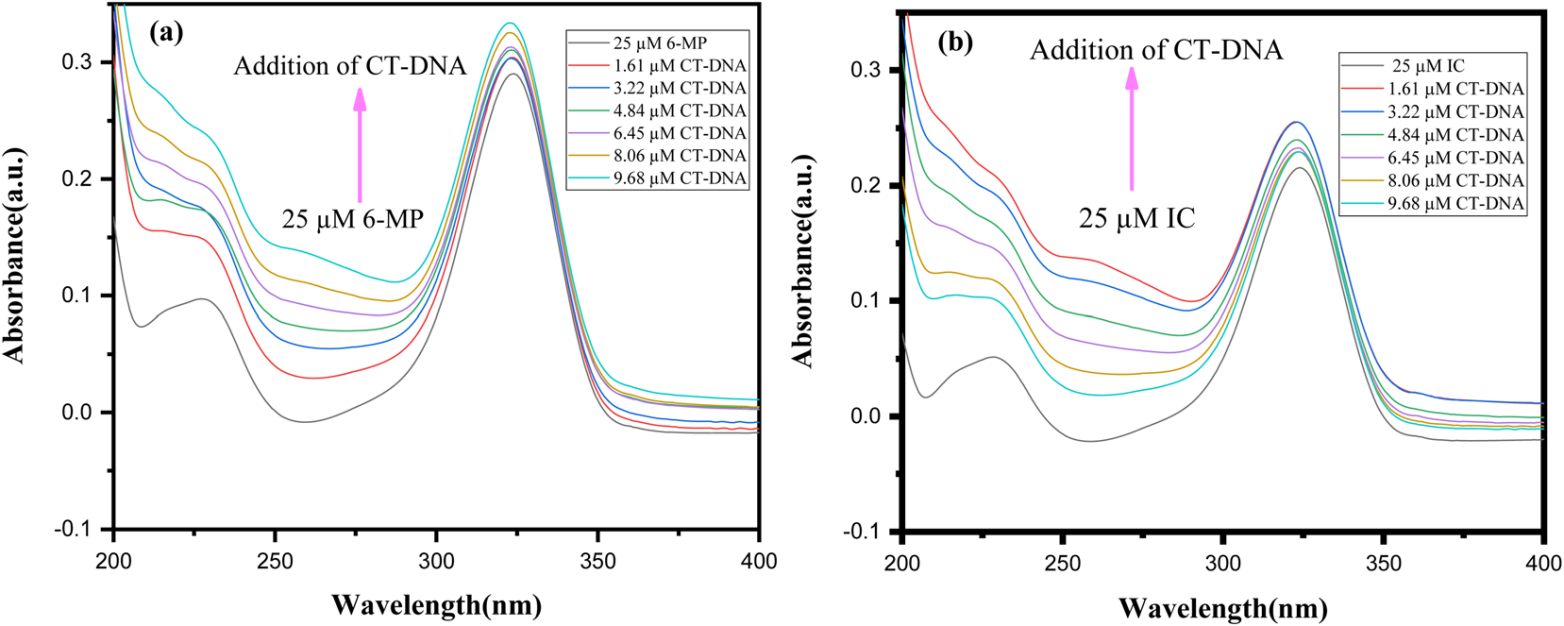
UV-Vis spectra of (a) 6-MP (b) IC at different concentrations of CT-DNA.

To cure or control the disease, many medications are already in use or in advanced clinical trials to target DNA.^86^ To manage diseases like cancer, these medications impede or change DNA function. Scientists aren't sure how medication molecules interact with DNA. As a result, DNA interaction investigations are intriguing and vital not only for understanding drug-DNA interaction mechanisms but also for finding novel and more effective DNA-targeted medicines. Our primary goal is to investigate how our synthesised 6-MP-CD IC interacts with DNA and how CD affects drug-DNA interactions, which could aid in the development of novel medications or boost the efficacy of existing ones.^15,23^ The UV spectra of a fixed concentration (25 μM) of drug and IC were collected with various CT-DNA concentrations, with a 30-minute incubation interval after each addition of CT-DNA (Fig. 13). The spectral data were used to produce a Benesi–Hildebrand plot using the following relation (6).^87^6
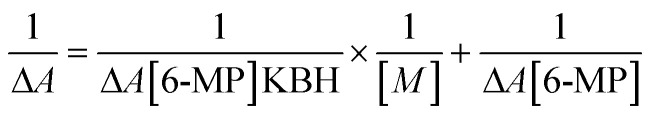
where [*M*] represents the DNA concentration, [Δ*A*] represents the change in absorbance at a specific wavelength, and Δ*A*[6-MP] represents the maximum absorbance. By graphing the reciprocal of the difference in absorbance with respect to the reciprocal of the DNA concentration, the Benesi–Hildebrand association constant (*K*_BH_) for complex formation was calculated from the ratio of the intercept to the slope.

In UV-Vis spectra, hyperchromic shift with modest hypsochromic shift was detected for both the drug and the IC, indicating intercalating binding mechanism with DNA (Fig. 13).^88^ The binding constants are listed in the table below (Table 7). The drug's DNA binding constant was found to be higher than the IC, which could be related to inclusion complex formation, which prevents the drug from intercalating with DNA. Again, in presence of a fixed concentration of CT-DNA, different 6-MP concentrations were also varied and summarised in Fig. S6.† As a result of this experiment, we may assume that in the presence of CT-DNA, the seeping of the guest molecule from the hollow of CDs into the aqueous solution is regular.^23^

**Table 7:** CT-DNA binding constant for different systems

Systems	*K*_BH_/M^−1^
6-MP	23.7 × 10^4^
(6-MP-β-CD)	7.4 × 10^4^

### 3.15 Antimicrobial effects of 6-MP in combination with β-CD

The results indicated the enhancement of the antimicrobial effect of 6-MP when used in combination with β-CD (as an inclusion complex) in the case of *E. coli* with the diameter of inhibition showing a 3-fold increase in the case of the IC as compared to 6-MP.^15^ β-CD and DMSO showed no effect on microbial growth (Fig. S7†). In the case of B, subtilis no antimicrobial effect of 6-MP was observed singly or when used as an IC with β-CD.

The differences in the structure of their cell walls can be held accountable for the differences in susceptibility of *Bacillus subtilis* and *E. coli*. *E. coli* is a Gram-negative bacteria having an outer lipid layer and a much lower proportion of peptidoglycan (a polymer of sugars and amino acids that form a mesh-like layer on the cell wall) as compared to the Gram-positive *Bacillus subtilis*.^89^

The outcome of this study is that the antimicrobial effect of 6-MP on *E. coli* can be enhanced when used in combination with β-CD as an inclusion complex whereas B-CD when used alone shows no antimicrobial effect (Table 8). To the best of our knowledge, this is the first report on the antimicrobial effect of 6-MP and further studies of its effect on other bacterial species along with elucidation of its mechanism of action can be conducted.

**Table 8:** Zone of inhibition observed against different bacteria (data represented as mean ± SD of triplicate determination)

Sample used	Zone of inhibition observed in *E. coli* (mm)	Zone of inhibition observed in *Bacillus subtilis* (mm)
6-MP	0.7 ± 0.2	0
β-CD	0	0
IC	2.0 ± 0.3	0
DMSO	0	0

### 3.16 Cytotoxicity effects on cancer cell line

**Fig. 14:**
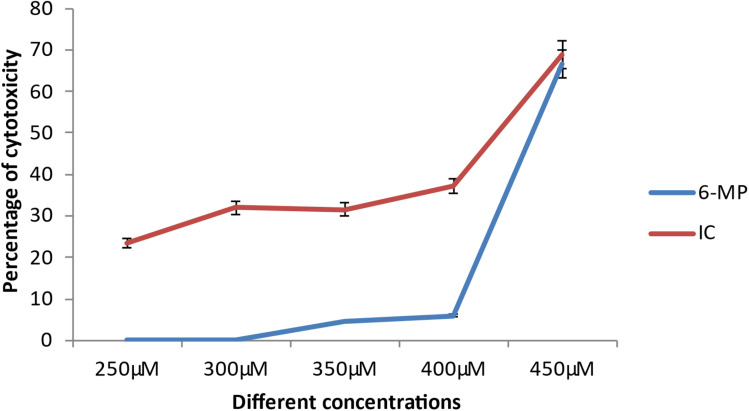
IC and 6-MP cytotoxicity percentages at various concentrations.

The cancer cell line's viability was reduced following medication therapy.^15^ The degree of cytotoxicity is similar to how much cellular viability is lost during pharmacological therapy. A drug's ability to limit cell reproducibility depends on how toxic it is. At lower dosages, the medication 6-MP and IC did not show any significant cytotoxicity against cancer cells (ACHN). However, IC-treated cells showed considerable cellular toxicity as drug concentration increased (from 250 μM on). The loss of cellular viability is likewise brought on by 6-MP, albeit at a higher concentration (350 μM) (Fig. 14). Comparing the inclusion complex treated cells to the control drug-treated cells reveals a large loss of cells.^15^ This finding suggests that the hazardous potential of the inclusion complex exceeds that of the control complex. Also, the IC_50_ value is much higher in the case of 6-MP (5.49 μM) than that of IC, *i.e.*, 4.18 μM. The inclusion complex is more harmful than the control medication, according to this finding. As a result, the aforementioned experimental data has demonstrated that this inclusion complex is effective anti-cancer medication for the treatment of renal carcinoma.

### 3.17 Theoretical study of inclusion phenomena

The Top and side views of the optimized geometries of the 6-MP-β-CD complex are illustrated in Fig. 6. In the relaxed geometry of the complex, drug 6-MP resides in the centre of the cavity *i.e.*, fully encapsulated by β-CD. The strong interaction of the 6-MP by β-CD has been confirmed by the residual short bond distances ranging from 2.16 to 3.22 Å. Hydrogen bonding interaction between the –NH group of 6-MP and –OH group of β-CD is responsible for showing very high adsorption energy (*E*_ads_ = −5.09 eV) in aqueous medium.

**Fig. 15:**
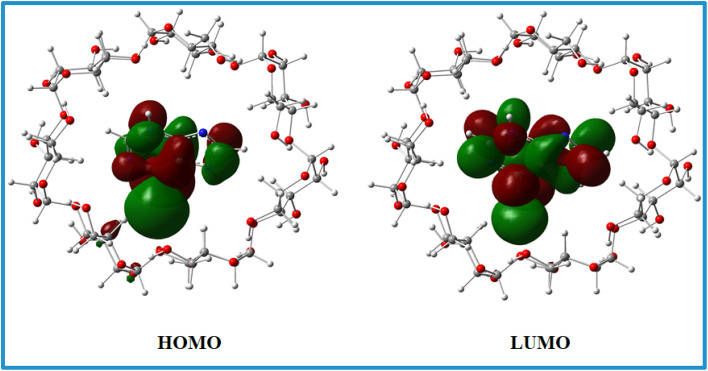
HOMO and LUMO charge densities of the 6-MP-β-CD complex.

HOMO and LUMO charge densities (Fig. 15) were analysed to understand the host–gust interaction and the amount of charge transfer occurring in these complex systems.^15,23^ We found that HOMO and LUMO densities are distributed mostly on the 6-MP indicating charge transfer is less prominent involving these complex systems.

**Fig. 16:**
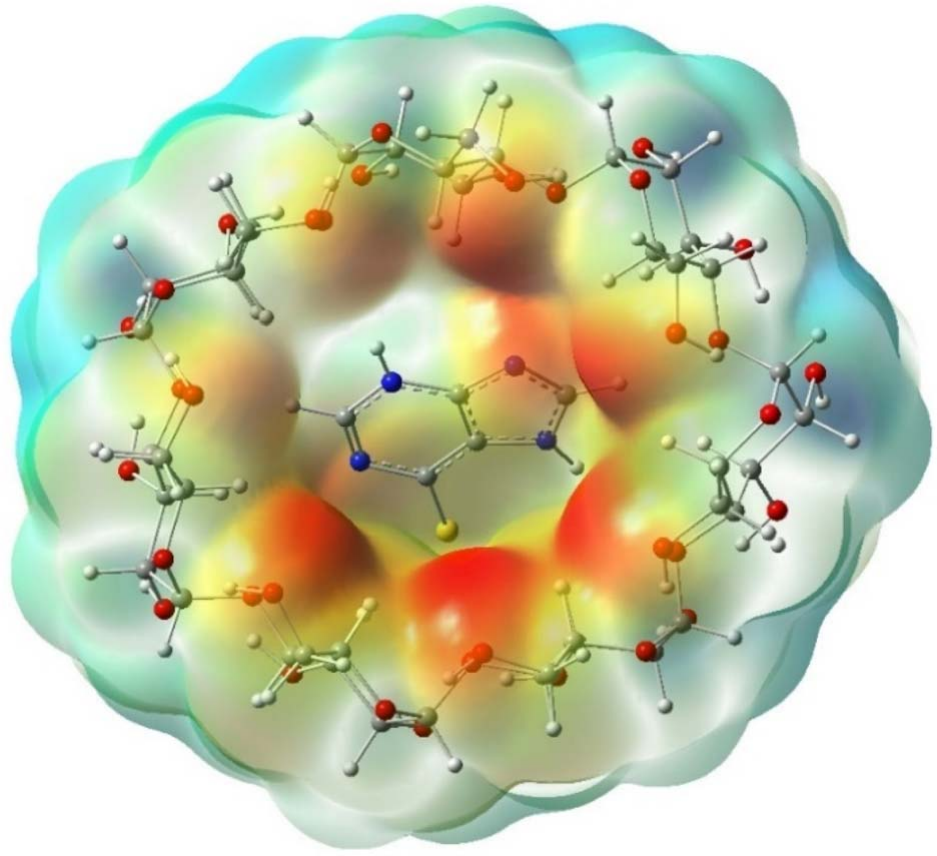
Electrostatic potential maps for the 6-MP-β-CD complex.

To understand the type of interactions between 6-MP and β-CD, we have analyzed the molecular electrostatic potential maps (ESP),^15,23^ as illustrated in Fig. 16. The red region of ESP maps around the β-CD cavity further signifies the stronger and prominent more interactions involving 6-MP and β-CD.

**Fig. 17:**
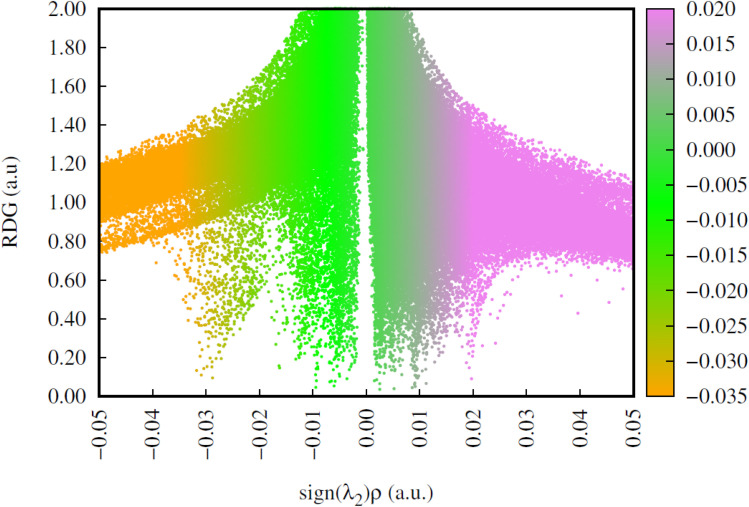
Plots of reduced density gradient (RDG) for 6-MP-β-CD complex.

To understand the week interactions operating 6-MP-β-CD we have analysed the RDG plots as shown in Fig. 17. We found that profound H-bonding interactions are operating between 6-MP and β-CD units as indicated by the greater scattered area of the negative region of the RDG plot (0.02–0.03 region).

## 4. Conclusion

The goal of our research was to synthesize a 6-MP-β-CD inclusion complex using a 1 : 1 molar ratio by a co-evaporation technique and its application in the field of medicinal chemistry. Spectroscopic analysis of the supramolecular interactions between the guest 6-MP and host β-CD was performed. ^1^H NMR, FTIR, PXRD, and UV-Vis spectroscopy were used to investigate the selective inclusion of 6-MP. The creation of a 1 : 1 molecular encapsulated complex is indicated by Job's plot and surface tension studies. The formation constants obtained from well-established procedures mandate the stability of IC formed, and the thermodynamic features reveal the truth about their creation capability. The aromatic component of 6-MP was inserted from the side of the broader rim of the cavity of β-CD, as demonstrated by ^1^H NMR spectroscopic analysis, and the significant changes in FTIR analysis support this. SEM image analysis has also validated the formation of IC and its surface figure. The insertion of medication 6-MP into the nanocavity of the β-CD improves the thermal stability of the IC up to 300 °C, according to a DSC and TGA investigation and the photostability experiments also supported the facts. In UV-Vis titration spectra of CT-DNA with various concentrations of pure 6-MP and IC, indicating intercalating binding mode with DNA. The regulated discharge of 6-MP molecules from the hydrophobic cavity to the polar aqueous media has been successfully described in the presence of CT-DNA, which may lessen toxicity inside the body. As a result, there's a good possibility that 6-MP and CT-DNA will attach similarly in the human body, and that the appropriate amount of 6-MP will be delivered to the desired location. An *in vitro* antibacterial test revealed that 6-MP's activity improved when it complexed with β-CD. The IC (IC_50_ = 4.18 μM) was found to have a considerable increase in *in vitro* cytotoxic activity against the human kidney cancer cell line (ACHN) compared to pure 6-MP (IC_50_ = 5.49 μM). Our theoretical calculations also show that the 6-MP-β-CD inclusion complex has higher adsorption energy (−5.09 eV), implying that it is more stable and this study also supports the experimental suggestion. As a result of the entire investigation, it can be stated that the production of the 6-MP-β-CD complex improves the solubility, stability and biological activity of 6-MP, which reduces the doses necessary in the human body and also leads to the development of anti-cancer drugs and the regulatory discharge of 6-MP from the hydrophobic cavity of β-CD into the aqueous solution provides a novel approach for a variety of applications and formulations in the food, medicinal, and pharmaceutical industries that requires no chemical modification.

## Author contributions

Mahendra Nath Roy, Modhusudan Mondal: conceptualization, resources. Mahendra Nath Roy: supervision. Modhusudan Mondal & Subhadeep Saha: writing—review & editing. Modhusudan Mondal, Shatarupa Basak & Narendra Nath Ghosh: investigation, writing—original draft, methodology, software, formal analysis. Modhusudan Mondal, Shatarupa Basak, Debadrita Roy, Biswajit Ghosh, Salim Ali, Khusboo Lepcha: validation, investigation, visualisation.

## Conflicts of interest

There were no known financial or personal conflicts of interest amongst the authors that could have influenced the work provided in this publication.

## Supplementary Material

RA-012-D2RA05072B-s001
