# Supplementary material for: Exploring inclusion complex of an anti-cancer drug (6-MP) with β-cyclodextrin and its binding with CT-DNA for innovative applications in anti-bacterial activity and photostability optimized by computational study
Source: RSC Adv. 2022 Oct 28;12(48):30936–51. doi: 10.1039/d2ra05072b (PMC9614615; doi:10.1039/d2ra05072b)
Supplement: RA-012-D2RA05072B-s001 [file RA-012-D2RA05072B-s001.pdf]

Electronic Supplementary Material (ESI) for RSC Advances.
This journal is © The Royal Society of Chemistry 2022

Supporting information

Exploring Inclusion complex of an anti-cancer drug (6-MP) with β-Cyclodextrin and its binding with CT-DNA for innovative applications in anti-bacterial activity and photostability optimized by computational study

Modhusudan Mondal [a], Shatarupa Basak[a], Salim Ali[a], Debadrita Roy[a], Subhadeep Saha[b], Biswajit Ghosh[a] , Narendra Nath Ghosh[c], Khusboo Lepcha[d], Kanak Roy[e] and Mahendra Nath Roy*[a,f]

[a] *Department of Chemistry, University of North Bengal, Darjeeling-734013, India*

[b] *Department of Chemistry, Government General Degree College, Pedong, Kalimpong-734311, India*

[c] *Department of Chemistry, University of Gour Banga, Mokdumpur, Malda-732103, India*

[d]*Department of Microbiology, University of North Bengal, Darjeeling-734013, India*

[e]*Department of Chemistry, Alipurduar University, Alipurduar-736121, India*

[f]*Vice-Chancellor, Alipurduar University, Alipurduar-736121, India*

**Corresponding author: Mahendra Nath Roy, Department of Chemistry, University of North Bengal, Darjeeling-734013, E-mail: mahendraroy2002@yahoo.co.in / vcapdu@gmail.com*

Table S1. Descriptions of the Chemicals used in the work.

Chemical Name	Source	Molecular weight(g/mol)	CAS Number	Mass fraction purity
6-Mercaptopurine Monohydrate	TCI, India	152.18(Anh)	6112-76-1	≥0.99
β-Cyclodextrin	Sigma-Aldrich, USA	1134.98	7585-39-9	≥0.99
Distilled water	Laboratory made	-	-	-

Table S2. Data for the Job plot performed by UV-Vis spectroscopy for 6-MP+β-CD system at 298.15K[a]

**6-MP + BETA - CYCLODEXTRIN**							
**6-MP(mL)**	**β-CD (mL)**	**6-MP(µM)**	**β-CD (µM)**	**[6-MP]/([6-MP]+[β-CD])**	**Absorbance (A)**	**ΔA**	**ΔA×[6-MP]/([6-MP]+[β-CD])**
0	3	0	100	0	0.0000	1.9695	0.0000
0.3	2.7	10	90	0.1	0.2196	1.7499	0.1750
0.6	2.4	20	80	0.2	0.4197	1.5498	0.3100
0.9	2.1	30	70	0.3	0.6164	1.3531	0.4059
1.2	1.8	40	60	0.4	0.8145	1.1550	0.4620
1.5	1.5	50	50	0.5	1.0116	0.9579	0.4789
1.8	1.2	60	40	0.6	1.2072	0.7623	0.4574
2.1	0.9	70	30	0.7	1.4195	0.5500	0.3850
2.4	0.6	80	20	0.8	1.5985	0.3710	0.2968
2.7	0.3	90	10	0.9	1.7827	0.1868	0.1681
3	0	100	0	1	1.9695	0.0000	0.0000

[a]Standard uncertainties in temperature (T) = ±0.01 K.

Table S3. Data for the surface tension study of 6-MP+β-CD system (concentration of stock solution of 6-MP = 10 mM, concentration of stock solution of β-CD = 10 mM) at 298.15K[a].

Volm of β-CD (mL)	Total volume (mL)	Conc of 6-MP (mM)	Conc of β-CD (mM)	ST (mN m^{-1})
0	10	10.000	0.000	41.1
1	11	9.091	0.909	42.4
2	12	8.333	1.667	43.8

3	13	7.692	2.308	44.8
4	14	7.143	2.857	45.6
5	15	6.667	3.333	46.4
6	16	6.250	3.750	47.1
7	17	5.882	4.118	47.6
8	18	5.556	4.444	48.1
9	19	5.263	4.737	48.5
10	20	5.000	5.000	48.8
11	21	4.762	5.238	49
12	22	4.545	5.455	49.2
13	23	4.348	5.652	49.2
14	24	4.167	5.833	49.2
15	25	4.000	6.000	49.3
16	26	3.846	6.154	49.3
17	27	3.704	6.296	49.4
18	28	3.571	6.429	49.4
19	29	3.448	6.552	49.4
20	30	3.333	6.667	49.5

[a]Standard uncertainties in temperature (T) = ±0.01 K.

Table S4. Data for the Benesi-Hildebrand double reciprocal plot performed by UV-Vis spectroscopy for 6-MP+*β*-CD system.

Temp /K[a]	[6-MP] /μM	[β-CD] /μM	Ao	A	ΔA	1/[β-CD] /M^{-1}	1/ΔA	Intercept	Slope	K_a /M^{-1}
298.15	40	10	0.8115	0.8149	0.0033	100000	295.9571	0.0027	21.525	7.98*10^3
	40	20		0.8193	0.0078	50000	128.0078			
	40	30		0.8202	0.0087	33333	114.2240			
	40	40		0.8220	0.0104	25000	95.4769			
	40	50		0.8241	0.0126	20000	79.2275			

	40	60		0.8247	0.0132	16667	75.5485			
	40	70		0.8279	0.0163	14286	61.0755			
	40	80		0.8299	0.0184	12500	54.1997			
	40	90		0.8314	0.0199	11111	50.0944			
	40	100		0.8359	0.0243	10000	41.0136			

[a]Standard uncertainties in temperature (T) = 0.01 K.

Table S5. Frequencies at FTIR spectra of 6-MP, β-CD and IC.

	Wave Number / cm^{-1}	Group
6-MP	3096.50	Stretching of C-H
	1666.00	Stretching of C=C
	1342.00	Stretching of C-N
	3425.60	Stretching of N-H
	1272.89	Stretching of –C=S
β-Cyclodextrin	3288.72	stretching of O-H
	2906.90	stretching of –C-H from –CH_2
	1417.80	bending of –C-H from –CH_2
	1152.50	bending of C-O-C
	1028.40	stretching of C-C-O
	937.38	skeletal vibration involving α-1,4linkage
6-MP-β-CD	3288.90	Stretching of –O-H of β-CD
	2904.20	Stretching of –C-H from –CH_2 of β-CD
	1613.90	Stretching of C=C
	1344.40	Stretching of C-N
	1150.00	Stretching of –C=S

	1022.20	Stretching of –C-C-O of β-CD

(a)

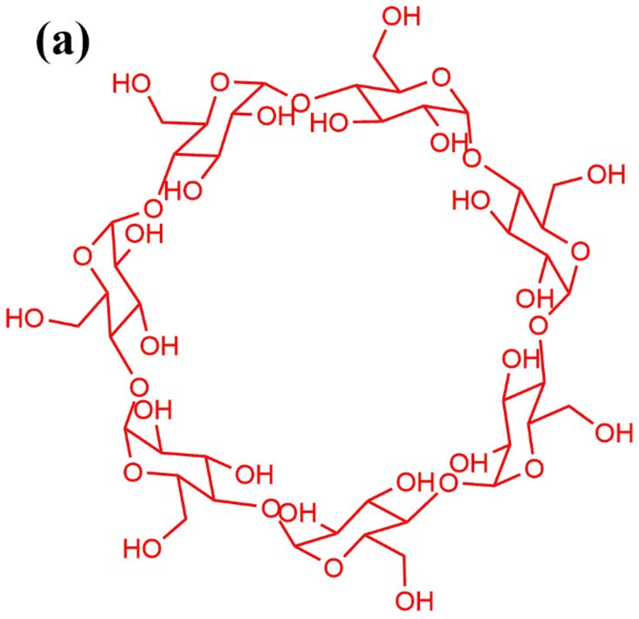

(b)

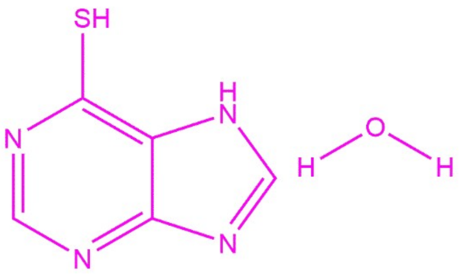

(a)

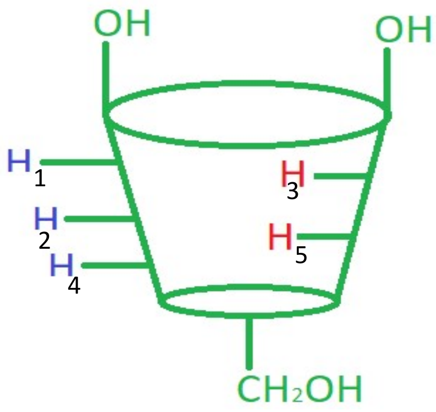

Figure S1. Molecular structures of (a) Beta Cyclodextrin and (b) 6-Mercaptopurine monohydrate.

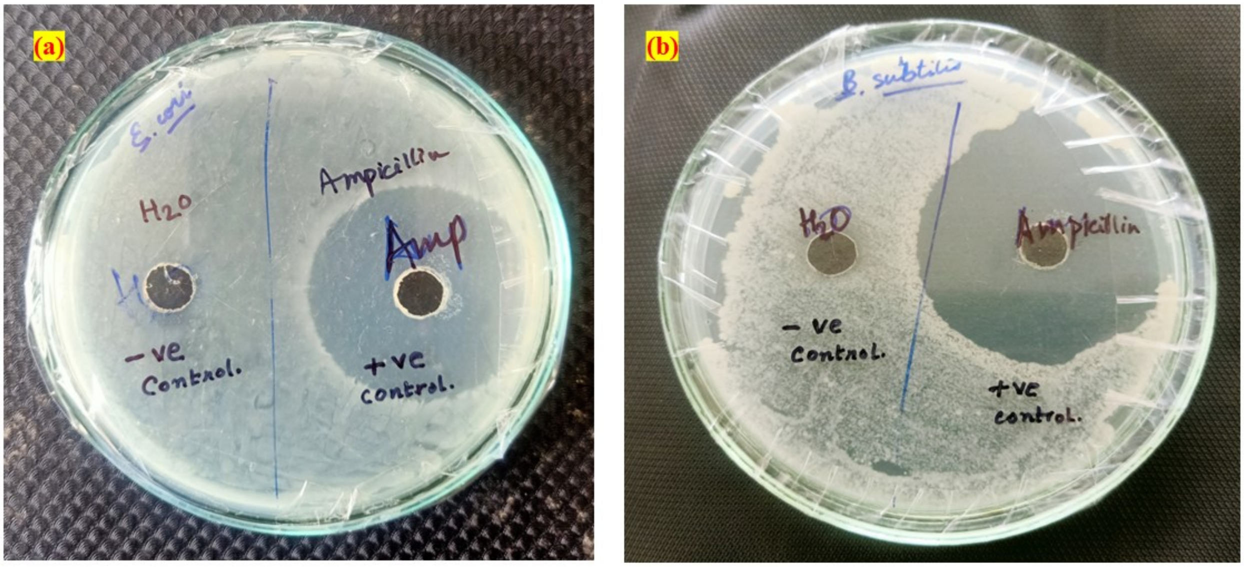

Figure S2: Pictures with Ampicillin as positive controls and sterile distilled water as negative controls in case of (a) *E.Coli*. and (b) *B. subtilis*.

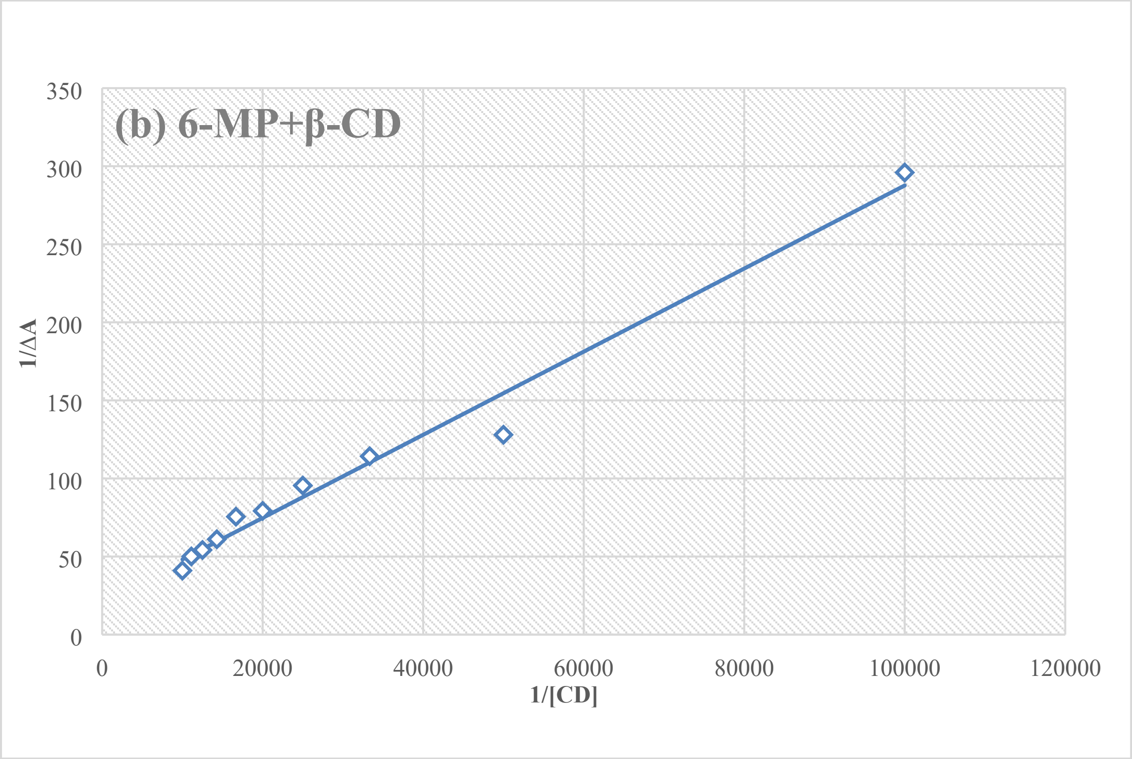

Figure S3. Benesi-Hildebrand double reciprocal plot for the effect of β-CD on the absorbance of TZ (324 nm) at 298.15 K (X axis = 1/[CD] and Y axis = 1/ΔA).

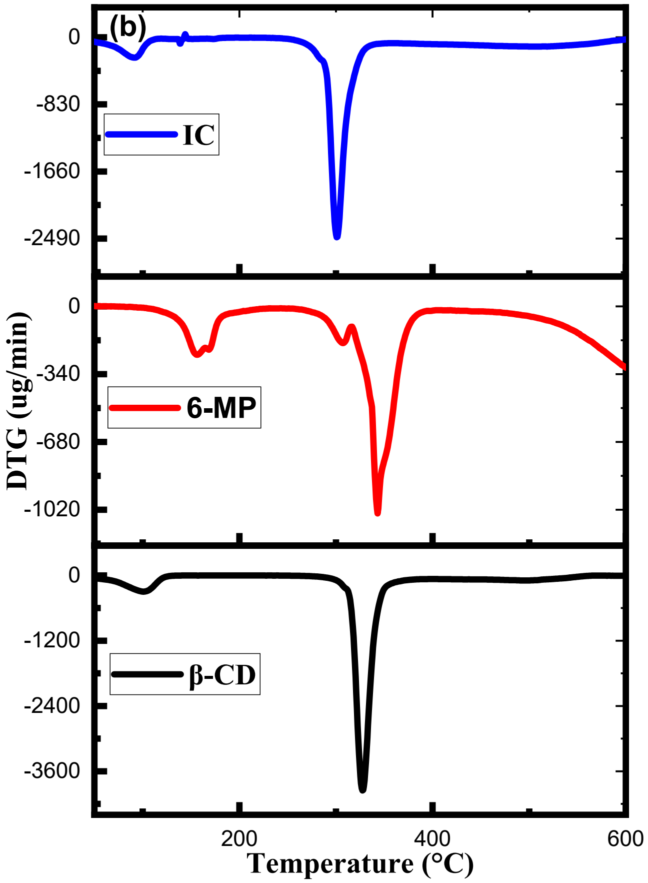

Figure S4. DTG thermograms of β-CD, 6-MP and its IC.

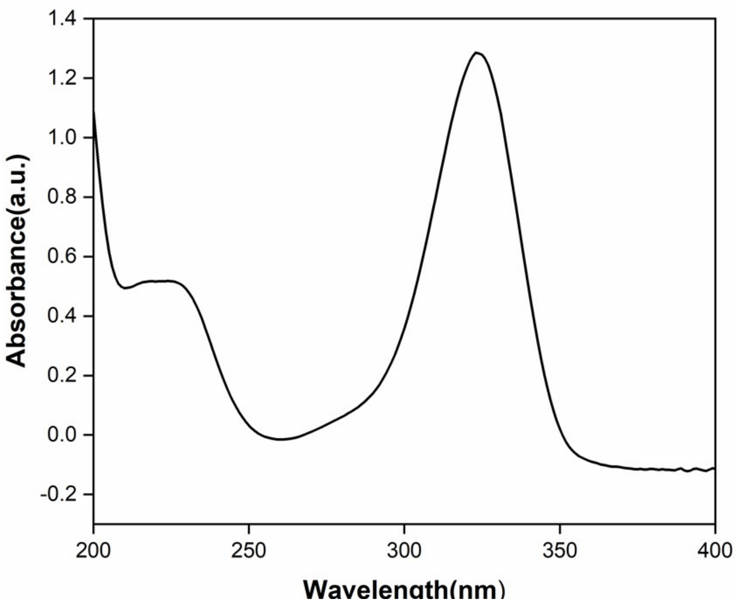

Figure S5. UV-Vis spectra of saturated concentration of IC.

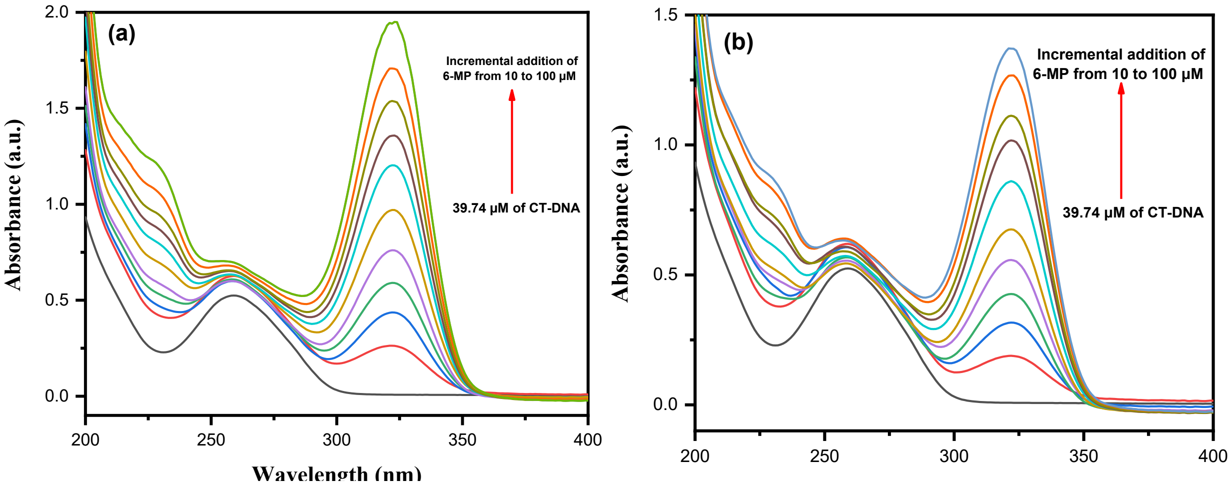

Figure S6. UV-Vis spectra of CT-DNA in presence of different concentration of 6-MP.

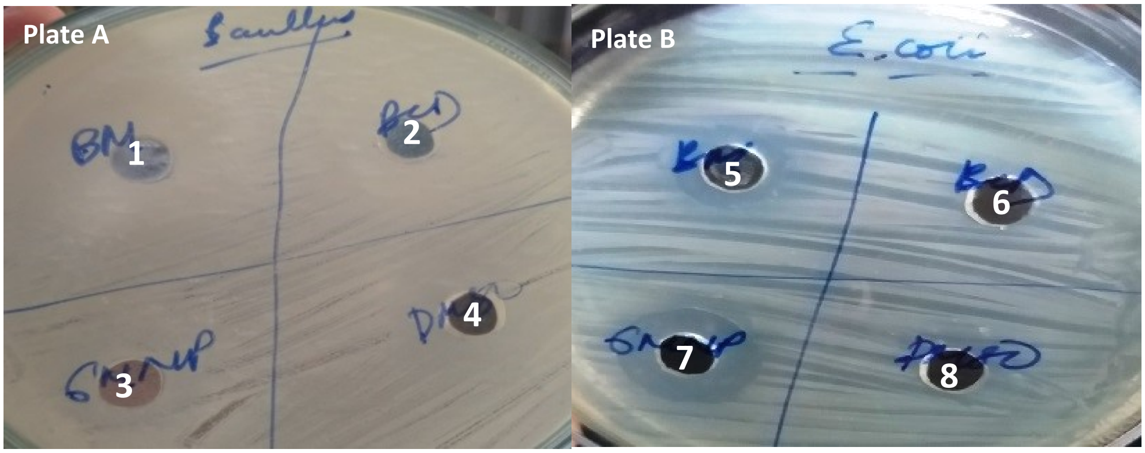

Figure S7: Well diffusion assay of 6-MP and β-CD, singly and in combination together as IC.

(Plate A shows susceptibility of *B. subtilis* towards 6-MP and β-CD, singly and in combination together as IC where Well1 contains 75 mM of 6-MP, Well 2 contains 75 mM of β-CD, Well 3 contains 75 mM of IC and Well 4 contains DMSO.)

(Plate B shows susceptibility of *E.coli* towards 6-M and β-CD, singly and in combination together as IC where Well 5 contains 75 mM of 6-MP,Well 6 contains 75 mM of β-CD, Well 7 contains 75 mM of IC and Well 8 contains DMSO.)
